# An oncolytic virus expressing a full-length antibody enhances antitumor innate immune response to glioblastoma

**DOI:** 10.1038/s41467-021-26003-6

**Published:** 2021-10-08

**Authors:** Bo Xu, Lei Tian, Jing Chen, Jing Wang, Rui Ma, Wenjuan Dong, Aimin Li, Jianying Zhang, E. Antonio Chiocca, Balveen Kaur, Mingye Feng, Michael A. Caligiuri, Jianhua Yu

**Affiliations:** 1 Department of Hematology and Hematopoietic Cell Transplantation, City of Hope National Medical Center, Los Angeles, CA USA; 2 Department of Immuno-Oncology, Beckman Research Institute, City of Hope Comprehensive Cancer Centre, Los Angeles, CA USA; 3 Pathology Core of Shared Resources Core, Beckman Research Institute, City of Hope National Medical Center, Los Angeles, CA USA; 4 Department of Computational and Quantitative Medicine, City of Hope National Medical Center, Los Angeles, CA USA; 5 Department of Neurosurgery, Brigham and Women’s Hospital and Harvey Cushing Neuro-oncology Laboratories, Harvard Medical School, Boston, MA USA; 6 The Vivian L. Smith Department of Neurosurgery, Mc Govern Medical School, University of Texas, University of Texas Health Science Center at Houston, Houston, TX USA; 7 Comprenhensive Cancer Center, City of Hope, Los Angeles, CA USA; 8 Hematologic Malignancies Research Institute, City of Hope National Medical Center, Los Angeles, CA USA

**Keywords:** Cancer microenvironment, Immunotherapy, CNS cancer, Cancer immunotherapy

## Abstract

Oncolytic herpes simplex virus-1 is capable of lysing tumor cells while alerting the immune system. CD47, in collaboration with SIRPα, represents an important immune checkpoint to inhibit phagocytosis by innate immune cells. Here we show locoregional control of glioblastoma by an oncolytic herpes virus expressing a full-length anti(α)-human CD47 IgG1 or IgG4 antibody. The antibodies secreted by the virus-infected glioblastoma cells block the CD47 ‘don’t eat me’ signal irrespective of the subclass; however, αCD47-IgG1 has a stronger tumor killing effect than αCD47-IgG4 due to additional antibody-dependent cellular phagocytosis by macrophages and antibody-dependent cellular cytotoxicity by NK cells. Intracranially injected αCD47-IgG1-producing virus continuously releases the respective antibody in the tumor microenvironment but not into systemic circulation; additionally, αCD47-IgG1-producing virus also improves the survival of tumor-bearing mice better than control oncolytic herpes virus combined with topical αCD47-IgG1. Results from immunocompetent mouse tumor models further confirm that macrophages, and to a lesser extent NK cells, mediate the anti-tumor cytotoxicity of antibody-producing oncolytic herpesviruses. Collectively, oncolytic herpes simplex virus-1 encoding full-length antibodies could improve immune-virotherapy for glioblastoma.

## Abstract (web summary)

Oncolytic herpes simplex virus-1 lyses cancer cells while increases their immunogenicity. Blocking the CD47 “don’t eat me” signal on cancer cells promotes their phagocytosis by macrophages. Authors here show that oncolytic viruses expressing anti-CD47 antibodies improve glioblastoma survival in mouse models.

## Introduction

Glioblastoma (GBM) is the most common and aggressive primary malignant brain tumor. GBM patients who undergo the standard of care, including surgical resection, chemotherapy, and radiotherapy, have a median survival of approximately 15 months^1^. Due to the early infiltrative nature of the disease, a complete surgical resection of GBM is largely unachievable. The intrinsic resistance of GBM to chemotherapy and radiotherapy also contributes to poor clinical outcomes. Given these attributes, new therapeutic agents are urgently needed for improving the outcome of GBM patients.

Oncolytic virus (OV) provides a potentially unique therapeutic approach for GBM as its mechanism of action is completely different from the standard approaches for the treatment of GBM. Oncolytic herpes simplex virus-1 (oHSV), one of the most widely investigated oncolytic viruses, is genetically engineered so that it can selectively lyse cancer cells while leaving normal cells largely intact. Like many other OVs, oHSV can also alert the patients’ immune system to attack tumor cells. Talimogene laherparepvec (Imlygic), the first FDA-approved oncolytic viral therapy, is based on the oHSV vector^2^. oHSV has proven to be relatively safe and has shown some activity in treating GBM^3^. In our previous studies, we found that oHSV treatment dramatically increased the intratumoral infiltration of immune cells^4–9^. However, tumor cells have evolved to engage the immune checkpoints, e.g., CD47 and PD-L1, and down-modulate the immune cells, thereby evading the antitumor immune response^10,11^. Thus, engineering oHSV to express a transgene(s) that could enhance immune responses and/or block engagement of immune checkpoints could be an effective approach to improve the overall efficacy of oHSV against GBM.

Tumor-associated macrophages (TAMs) are the major tumor-promoting immune cells in the GBM microenvironment^12,13^. Hence, re-education of TAMs in GBM may provide a promising antitumor strategy^14,15^. The CD47-signal regulatory protein alpha (SIRPα) pathway is one of the most studied phagocytosis checkpoints in macrophages^16^. CD47-SIRPα myeloid checkpoint blockade has been shown to effectively enhance tumor phagocytosis and reduce tumor burden^17–20^. A humanized anti-CD47 monoclonal antibody (mAb), which directly inhibits the CD47−SIRPα interaction, is currently in clinical trials^21^ and shows a strong activity against GBM in multiple murine models^18^. For safety concerns, this antibody was usually engineered on a human IgG4 scaffold to minimize Fc-dependent effector functions of innate immunity such as natural killer (NK) cell antibody-dependent cellular cytotoxicity (ADCC) and macrophage antibody-dependent cellular phagocytosis (ADCP)^22^. An IgG1 form of anti-CD47 antibody should possess ADCP and ADCC anti-GBM activity; however, infusion toxicities and difficulty in penetration through the blood−brain barrier (BBB) are current challenges that limit the systemic treatment of GBM with an IgG1 form of anti-CD47 antibody.

In this work, we generate an oHSV carrying a transgene encoding a full-length anti-CD47 antibody on a human IgG1 scaffold (termed OV-αCD47-G1) and the other as control carrying a transgene encoding a full-length anti-CD47 antibody on a human IgG4 scaffold (OV-αCD47-G4). Antibodies secreted by GBM cells infected with OV-αCD47-G1 or OV-αCD47-G4 enhance tumor phagocytosis by blocking the CD47−SIRPα axis, while the secreted αCD47-G1 instead of αCD47-G4 also induces Fc receptor-mediated tumor phagocytosis by macrophages and tumor cytotoxicity by NK cells. In vivo, intracranial (i.c.) administrations of OV-αCD47-G1 and OV-αCD47-G4 result in the continuous release of both antibodies specifically into the GBM tumor microenvironment, while OV-αCD47-G1 shows a superior effect and significantly prolongs survival of GBM-bearing mice when compared to OV-αCD47-G4 or their parental OV-Q1. Our findings indicate that engineering oHSV to express a full-length anti-CD47 antibody, especially the IgG1 version, with locoregional delivery is a promising and non-toxic approach to enhance oncolytic virotherapy of GBM.

## Results

### OV-αCD47-G1- or OV-αCD47-G4-infected GBM cells secret a full-length anti-CD47 antibody

**Fig. 1:**
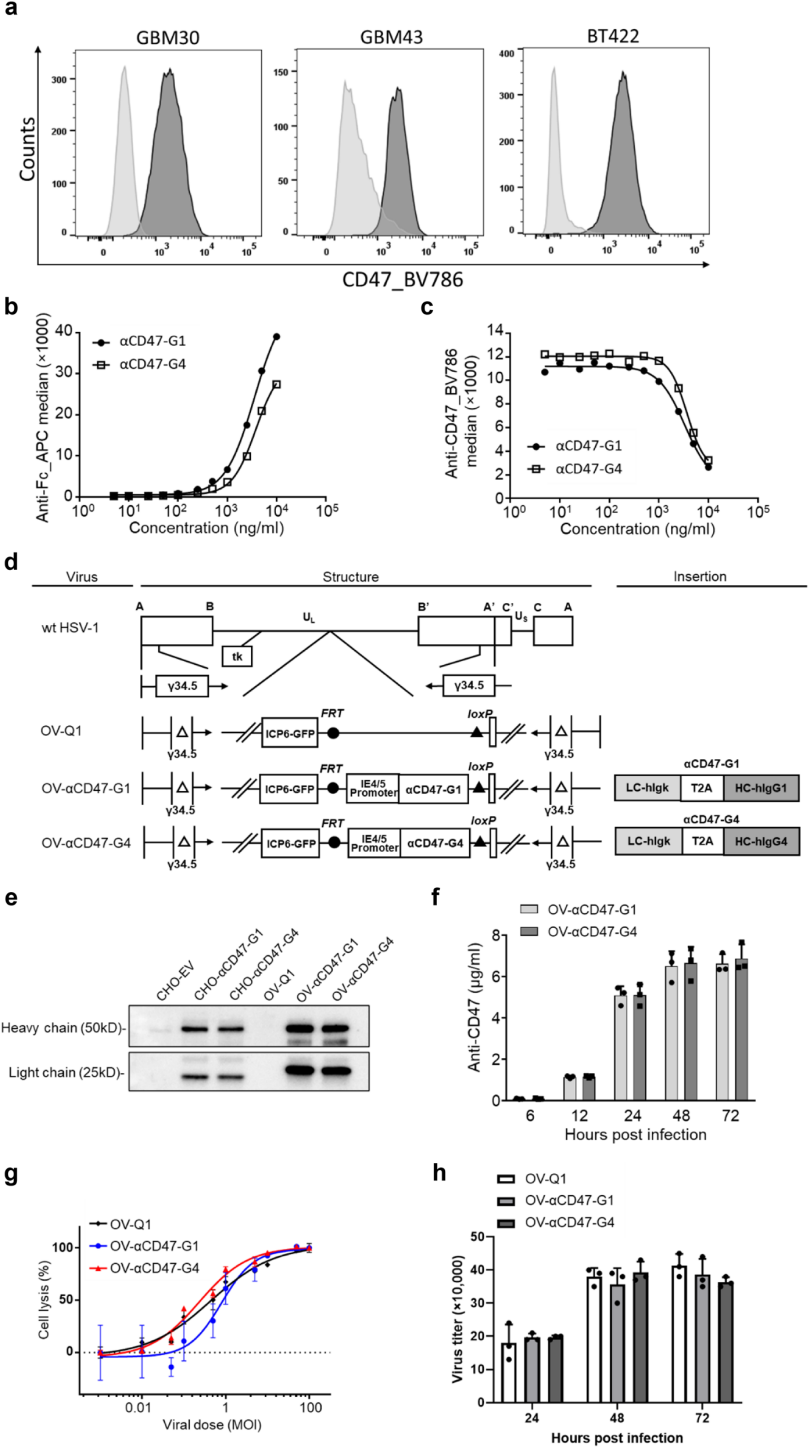
Construction and characterization of OV-Q1, OV-αCD47-G1, and OV-αCD47-G4. **a** Flow cytometric assay of CD47 expression on GBM patient-derived GBM30, GBM43, and BT422 cells. **b** Human CD47 binding affinity of αCD47-G1 and αCD47-G4 purified from CHO cells as assessed by flow cytometry. U251T2 human GBM cells were incubated with an increasing concentration of αCD47-G1 or αCD47-G4 mAbs followed by staining with an APC-conjugated anti-human Fc. **c **Inhibition of anti-CD47 mAb binding by αCD47-G1 and αCD47-G4 mAbs. U251T2 cells were incubated with an increasing concentration of αCD47-G1 or αCD47-G4 mAb followed by incubation with a conjugated anti-human CD47 antibody and assessed by flow cytometry. **d** Schematic of oncolytic viruses used in this study. Top: genetic map of wild-type HSV-1. Second: genetic map of control oHSV, OV-Q1, with deletion of two copies of γ34.5, dysfunction of ICP6, and insertion of the GFP gene. Third: genetic map of OV-αCD47-G1 showing the inserted coding gene of the IgG1 version of anti-CD47 (αCD47-G1). Fourth: genetic map of OV-αCD47-G4 showing the inserted coding gene of the IgG4 version of anti-CD47 (αCD47-G4). The light-chain and heavy-chain coding genes of αCD47-G1 and αCD47-G4 are linked by a T2A sequence and are driven by the viral pIE4/5 promoter. LC light chain, HC heavy chain, hIgκ human Igκ light chain, hIgG1 human IgG1 heavy chain, hIgG4 human IgG4 heavy chain. **e** Immunoblotting performed with concentrated supernatants of engineered CHO cells and oHSV-infected U251T2 human GBM cells by anti-human Fc or anti-human Igκ antibody. **f** αCD47-G1 and αCD47-G4 yields from supernatants of OV-αCD47-G1- and OV-αCD47-G4-infected U251T2 cells as determined by ELISA assay. **g** U251T2 human GBM cells were infected with OV-Q1, OV-αCD47-G1, or OV-αCD47-G4 at the indicated MOIs. Cell lysis was analyzed at 3 days after infection by CCK8 cell viability assay. **h** U251T2 cells were infected with OV-Q1, OV-αCD47-G1, or OV-αCD47-G4 at an MOI of 2. The supernatants were harvested at indicated time points for viral reproduction using a plaque assay in Vero cells. Experiments in (a−c) and (e−h) are representative of three independent experiments with similar data. Data are presented as mean values ± SD (*n* = 3).

We first assessed six human GBM cell lines, including patient-derived stem-like GBM30, GBM43 and BT422, and observed that each uniformly expressed CD47 on the cell surface (Fig. 1a and Supplementary Fig. 1). We then generated constructs of αCD47-G1 and αCD47-G4, an IgG1 or IgG4 version, respectively, of the humanized anti-human CD47 antibody (clone 5F9). αCD47-G4 was reconstructed as previously reported^22^. αCD47-G1 was constructed by replacing the human IgG4 constant region of αCD47-G4 with the human IgG1 constant region. CHO cells were transduced with lentiviruses carrying the corresponding coding genes of full-length heavy chain and light chain to produce αCD47-G1 and αCD47-G4 for functional tests. The results of CD47 binding assay showed a similar dose-dependent binding affinity of αCD47-G1 and αCD47-G4 to CD47(+) U251T2 GBM cells, demonstrating that the two antibodies have nearly equal binding affinity to the CD47(+) U251T2 cells (Fig. 1b). The results of CD47 blocking assay showed a similar dose-dependent CD47 blocking capacity of αCD47-G1 and αCD47-G4 (Fig. 1c). Next, we generated oHSVs expressing αCD47-G1 or αCD47-G4, using the parental OV-Q1, which is double-attenuated with an inactivated ribonucleotide reductase gene (ICP6) and deletions of both copies of the neurovirulence gene (ICP34.5) that limits its replication to tumor cells and reduces its neurovirulence^23,24^ (Fig. 1d). The corresponding light-chain and heavy-chain coding genes of αCD47-G1 or αCD47-G4 were linked with a DNA sequence encoding a T2A self-cleaving peptide and were inserted into the ICP6 locus of OV-Q1, driven by the promoter of the HSV-1 immediate early gene IE4/5 (Fig. 1d). The two oHSVs were generated in a manner that we previously reported^4^ and are termed OV-αCD47-G1 and OV-αCD47-G4. The genetic maps of wild-type human HSV-1, OV-Q1, OV-αCD47-G1, and OV-αCD47-G4 are illustrated in Fig. 1d. Infection of GBM cells with OV-αCD47-G1 or OV-αCD47-G4 enabled the infected cells to secrete αCD47-G1 or αCD47-G4, respectively. The supernatants from OV-αCD47-G1-, OV-αCD47-G4- and OV-Q1-infected U251T2 GBM cells as well as the αCD47-G1- and αCD47-G4-expressing CHO cells and the control CHO cells were collected for immunoblot assay, which showed that human IgG heavy chain and light chain existed within the supernatants from OV-αCD47-G1- and OV-αCD47-G4-infected U251T2 GBM cells (Fig. 1e). The light chain expressed by OV-αCD47-G1- and OV-αCD47-G4-infected U251T2 GBM cells displayed a higher molecular weight than that expressed by the corresponding CHO cells using a two-plasmid system, likely due to the remaining 17 amino acid N-terminus residues of the T2A peptide left in the light chain resulting from the self-cleavage of T2A between glycine (G) and proline (P) at its C-terminus in the GBM cells, as previously described^25^ (Fig. 1e). The yield of anti-CD47 antibody from OV-αCD47-G1- and OV-αCD47-G4-infected U251T2 GBM cells were quantified by enzyme-linked immunosorbent assay (ELISA), using corresponding antibodies purified from CHO cells with known concentrations as standards. The OV-αCD47-G1- and OV-αCD47-G4-infected U251T2 GBM cells released appreciable and similar amounts of anti-CD47 antibody as early as 12 h post infection (hpi), and the yields reached near-maximum levels over 5 µg/ml at 24 hpi and peak levels over 6 µg/ml in culture at 48 hpi (Fig. 1f). The reversed version of OV-αCD47-G1 (OV-αCD47-G1-HL) that switches the position of light-chain and heavy-chain coding gene was also constructed (Supplementary Fig. 2a). It appeared that OV-αCD47-G1-infected U251T2 GBM cells produced a slightly higher level of anti-CD47 antibody than OV-αCD47-G1-HL-infected GBM cells (Supplementary Fig. 2b), and thereafter all subsequent experiments were performed using OV-αCD47-G1 instead of OV-αCD47-G1-HL.

To test the oncolytic effect of OV-αCD47-G1 and OV-αCD47-G4, we infected the human GBM cell lines U251T2 and Gli36ΔEGFR with the corresponding viruses. We did not observe a substantial difference in cell death among GBM cells infected with OV-αCD47-G1, OV-αCD47-G4, or OV-Q1 (Fig. 1g and Supplementary Fig. 3a). We next evaluated the viral production capacity of OV-αCD47-G1 and OV-αCD47-G4. Results showed that OV-αCD47-G1- and OV-αCD47-G4-infected U251T2 GBM cells or Gli36ΔEGFR GBM cells produce similar amounts of virus when compared to the same GBM cells infected with OV-Q1 (Fig. 1h and Supplementary Fig. 3b). Therefore, engineering oHSV to express αCD47-G1 or αCD47-G4 does not affect their oncolytic ability and virus productivity.

### αCD47-G1 secreted by OV-αCD47-G1-infected GBM cells enhances phagocytosis and blocks “don’t eat me” signaling in macrophages

**Fig. 2:**
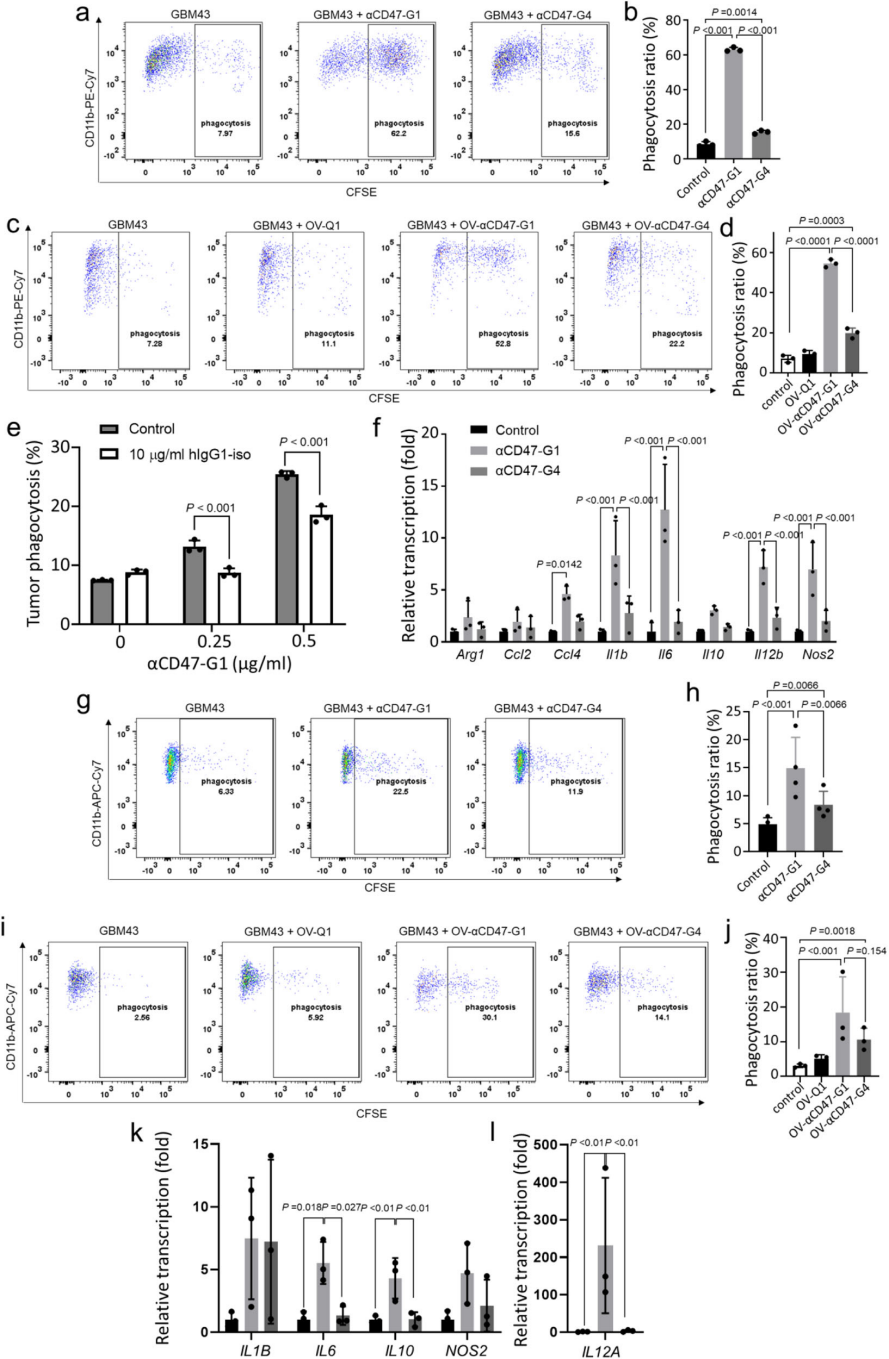
αCD47-G1 and αCD47-G4 induce phagocytosis of GBM cells. **a**, **b** GBM43 cells were labeled with CFSE and cocultured with mouse BMDMs at an effector:target ratio of 1:2 in the presence of vehicle control, CHO-derived αCD47-G1 or αCD47-G4 at the dose of 5 μg/ml. The percentage of BMDM phagocytosis against GBM43 cells (CD11b+CFSE+) was assayed by flow cytometry. **c**, **d** GBM43 cells were labeled with CFSE and cocultured with mouse BMDMs at an effector:target ratio of 1:2 in the presence of vehicle control, conditioned media from OV-Q1-, OV-αCD47-G1-, and OV-αCD47-G4-infected U251T2 cells. The percentage of BMDM phagocytosis against GBM43 cells (CD11b+CFSE+) was assayed by flow cytometry. **e** Phagocytosis assay performed with excess human IgG1 to block BMDM Fc receptors. BMDMs were incubated with vehicle control or human IgG1 at the dose of 10 µg/ml for 30 min prior to coculture with GBM43 cells in the presence of increasing αCD47-G1 for 2 h. The percentage of BMDM phagocytosis against GBM43 cells (CD11b+CFSE+) was assayed by flow cytometry. **f** BMDMs were cocultured with GBM43 cells at a ratio of 1:1 with or without αCD47-G1 or αCD47-G4 for 6 h, followed by gene transcript quantification by reverse transcription (RT) quantitative (q)PCR. **g**, **h** Effect of 5 μg/ml of αCD47-G1 and αCD47-G4 purified from transduced CHO cells on phagocytosis against GBM43 cells by primary human macrophages. **i**, **j** The effect of conditioned media from the culture of OV-αCD47-G1- and OV-αCD47-G4-infected U251T2 cells on phagocytosis against GBM43 cells by primary human macrophages. Phagocytosis was assayed by flow cytometry. **k**, **l** Primary human macrophages were cocultured with GBM43 cells at a ratio of 1:1 with or without αCD47-G1 or αCD47-G4 for 6 h, followed by gene transcript quantification by RT-qPCR. All experiments were performed at least with three human donors or mice. Error bars represent standard deviations of means of different donors. For (**b**), (**d**−**f**), (**h**), and (**j**−**l**), one-way ANOVA with *P* values corrected for multiple comparisons by the Bonferroni test.

We next compared the ability of αCD47-G1 and αCD47-G4 to modulate phagocytosis of human GBM cells by murine and human macrophages. Previous studies showed that SIRPα on murine macrophages can bind to human CD47 to stimulate “don’t eat me” signaling^26^. We thus started with mouse bone-marrow-derived macrophages (BMDMs) isolated from BALB/c mice as effector cells, as previously reported by other groups^27–29^. Flow cytometry results showed that both αCD47-G1 and αCD47-G4 purified from transduced CHO cells induced a significantly higher level of BMDMs phagocytosis against patient-derived GBM43 cells (Fig. 2a, b) and BT422 cells (Supplementary Fig. 4a, b) compared to vehicle control. The phagocytosis assays were also repeated with the unconcentrated supernatants from OV-αCD47-G1-, OV-αCD47-G4-, or OV-Q1-infected U251T2 GBM cells. Consistent with the above results, αCD47-G1 from the infected cells has over a two-fold higher level of macrophage phagocytosis against GBM cells compared to αCD47-G4 from the infected cells (Fig. 2c, d). To prove that αCD47-G1 induces GBM phagocytosis via ADCP, we repeated the phagocytosis assay with Fc receptor blockade. Pre-incubating BMDMs with high-dose (10 µg/ml) isotype human IgG1, which competes with αCD47-G1 to bind to Fc receptors, significantly inhibited αCD47-G1-induced GBM phagocytosis by BMDMs (Fig. 2e). We also found that αCD47-G1 but not αCD47-G4 dramatically activated transcription of typical macrophage cytokine genes of mouse BMDMs that were previously reported to respond to IgG1 antibody^30^, such as *Il1b, Il6, Il10, Il12b* and *Nos2* (Fig. 2f).

The effects of αCD47-G1 and αCD47-G4 on inducing the human macrophage phagocytosis were also tested. For this purpose, primary human donor-derived macrophages were used as effector cells, as previously reported by other groups^31^. Similar to the results as we collected using mouse BMDMs, compared to vehicle control, both αCD47-G1 and αCD47-G4 purified from transduced CHO cells significantly enhanced the phagocytosis of GBM43 cells by human macrophages. However, the effect of the former is more substantial than the latter due to having an additional strong ADCP effect (Fig. 2g, h). Similar results were found by using the unconcentrated supernatants from OV-αCD47-G1-, OV-αCD47-G4- or OV-Q1-infected U251T2 GBM cells. Both αCD47-G1 secreted by OV-αCD47-G1-infected cells and αCD47-G4 secreted by OV-αCD47-G4-infected cells significantly enhanced the phagocytosis of GBM43 cells by human macrophages, although the effect of the former is more substantial than the latter (Fig. 2i, j). However, when using CD47-knockout GBM43 cells generated by CRISPR/Cas9 gene editing (GBM43ΔCD47 cells) as target cells, αCD47-G1 and αCD47-G4 treatment were not able to enhance the macrophage phagocytosis (Supplementary Fig. 5). αCD47-G1 also activated the transcription of typical macrophage cytokine genes of human macrophages, such as *IL1B, IL6, IL10, NOS2* (Fig. 2k), and *IL12A* (Fig. 2l).

For both murine and human macrophages, the increased phagocytosis effect of αCD47-G1 above vehicle control should be from both blockade of “don’t eat me” signaling and ADCP while the effect above αCD47-G4 should be from ADCP only, as IgG1 rather than IgG4 antibodies can induce substantial ADCP via an Fc receptor-mediated effect, likely due to the distinct physical properties of IgG1 and IgG4 antibodies^32^.

### αCD47-G1 secreted by OV-αCD47-G1-infected GBM cells induces potent NK cell-mediated ADCC

**Fig. 3:**
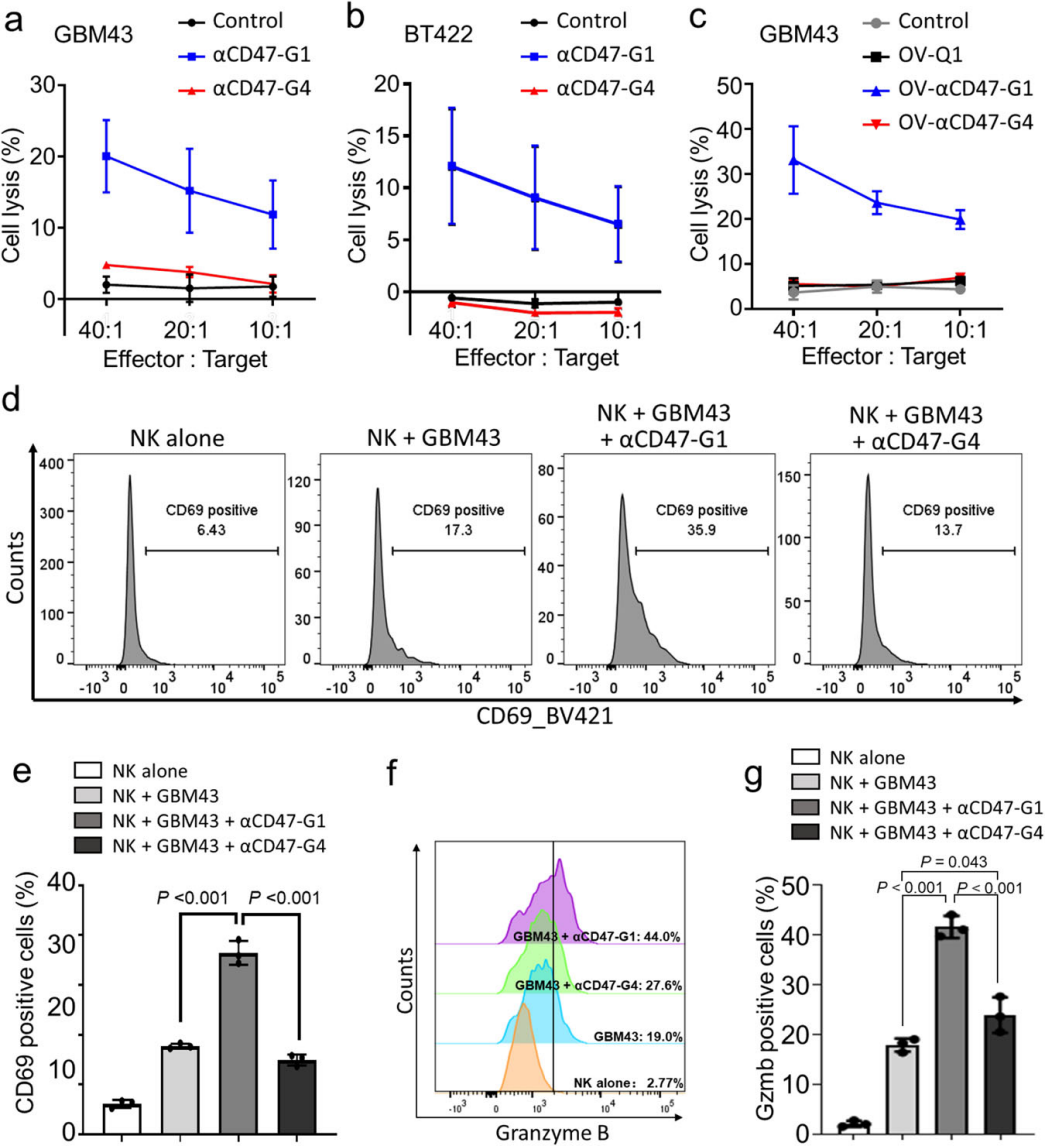
αCD47-G1 but not αCD47-G4 induces cytotoxicity of human NK cells against GBM cells. **a**, **b** Cytotoxicity of human primary NK cells against αCD47-G1- and αCD47-G4-treated (**a**) GBM43 and (**b**) BT422 human GBM cells. **c** Cytotoxicity of human primary NK cells against GBM43 cells in control media or conditioned media from the culture of OV-Q1-, OV-αCD47-G1- or OV-αCD47-G4-infected U251T2 GBM cells. **d** The effect of αCD47-G1 or αCD47-G4 on the expression of the NK cell activation marker CD69 when cocultured with GBM43 cells at an effector:target ratio of 1:1. **e** Summary data of (**d**). All experiments were performed with three donors in triplicate. **f** The effect of αCD47-G1 and αCD47-G4 on granzyme B expression of NK cells when cocultured with GBM43 cells. **g** Summary data of (**f**). All experiments were performed with three donors in triplicate. Error bars represent standard deviations of means of three donors. For (**e**) and (**g**), one-way ANOVA with *P* values corrected for multiple comparisons by the Bonferroni test.

NK cells play a critical antitumor activity in cancers, including GBM, via natural cytotoxicity and ADCC, especially in combination with antibody therapy^33^. In order to determine how αCD47-G1 or αCD47-G4 affects NK cell antitumor activity, we conducted a standard ^51^Cr release assay by co-culturing human NK cells with human GBM patient-derived tumor cells GBM43 and BT422 labeled with ^51^Cr in the presence or absence of αCD47-G1 or αCD47-G4 purified from lentivirus-transduced CHO cells. The results showed that αCD47-G1 but not αCD47-G4 induced strong NK cell cytotoxicity (Fig. 3a, b and Supplementary Fig. 6). Consistent data were collected when GBM cell lines U251T2, Gli36ΔEGFR, LN229, and patient-derived GBM30 cells were used as target cells (Supplementary Fig. 7a, b). Then we repeated this experiment with the unconcentrated supernatants from OV-αCD47-G1-, OV-αCD47-G4-, and OV-Q1-infected U251T2 GBM cells. GBM43 cells incubated with the unconcentrated supernatants from OV-αCD47-G1-infected U251T2 GBM cells underwent substantially more cytolysis compared to those incubated with the supernatants from OV-Q1-infected or OV-αCD47-G4-infected U251T2 cells (Fig. 3c). No differences were detected between the supernatants from OV-Q1- and OV-αCD47-G4-infected U251T2 GBM cells (Fig. 3c). However, for GBM43ΔCD47 cells, neither αCD47-G1 nor αCD47-G4 treatments induced stronger NK cell cytotoxicity (Supplementary Fig. 7c). NK cell activation induced by αCD47-G1 was also confirmed by measuring the expression of the activation marker CD69 and granzyme B by flow cytometry. The results showed that compared to vehicle control, αCD47-G1 but not αCD47-G4 purified from transduced CHO cells significantly increased the surface expression of CD69 on NK cells (Fig. 3d, e and Supplementary Fig. 7d, e). Compared to vehicle control, αCD47-G1 significantly increased the expression of granzyme B, while αCD47-G4 showed a slight increase, in NK cells (Fig. 3f, g). The results of IFN-γ ELISA assay showed that compared to vehicle control, αCD47-G1 and αCD47-G4 both failed to induce IFN-γ secretion, even in the presence of IL-2, IL-12, IL-15, or IL-18 (Supplementary Fig. 8).

### Comparison of systemic i.p. injection versus locoregional OV delivery of anti-CD47 antibody

**Fig. 4:**
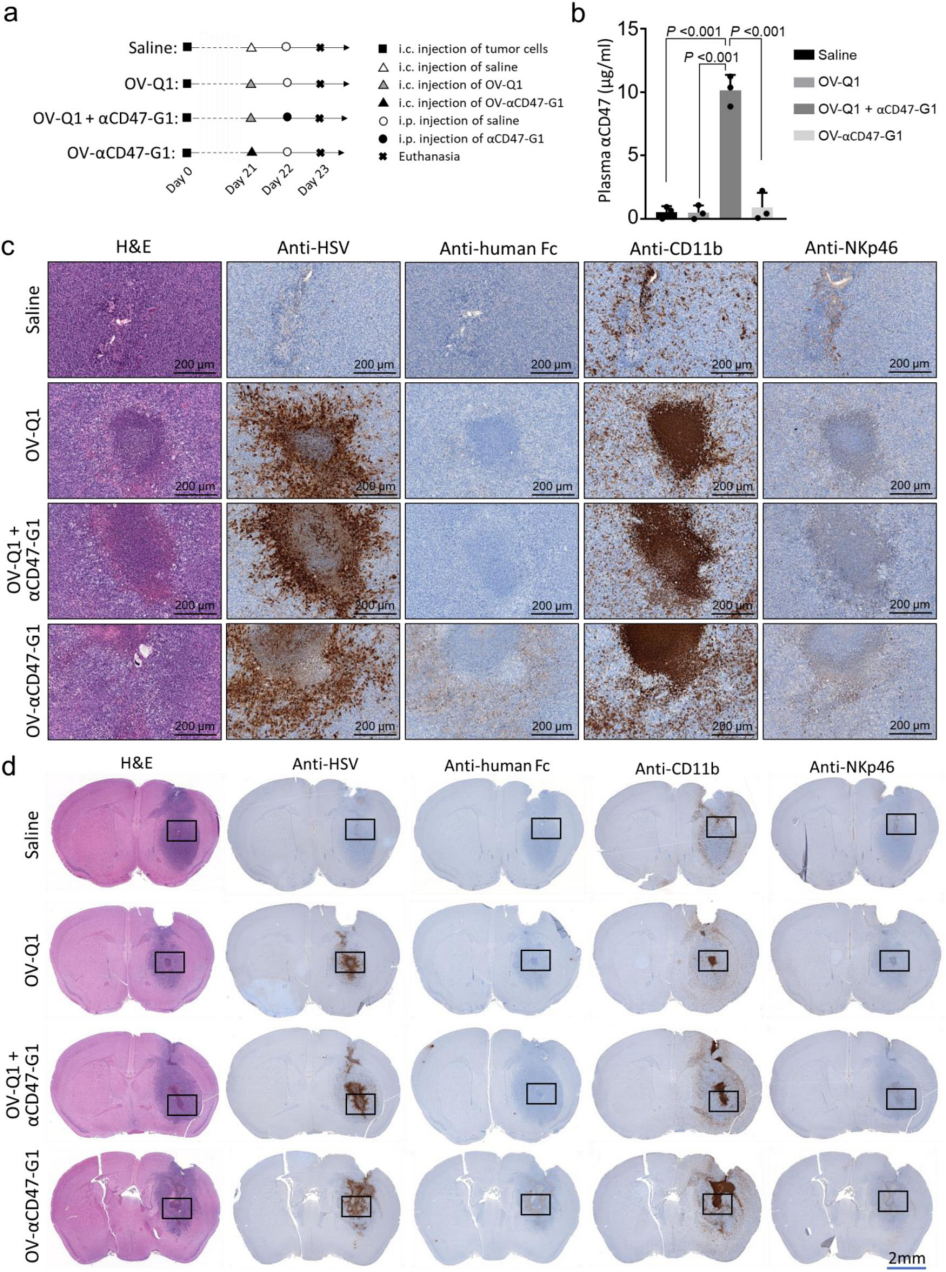
OV-αCD47-G1 is effective for locoregional delivery of the anti-CD47 antibody, while systemic administration of αCD47-G1 is not effective. **a** Experimental timeline for in vivo studies using an orthotopic model of human GBM. Experiment details are provided in the main text. Group 1, saline; Group 2, OV-Q1; Group 3, a combination of OV-Q1 plus i.p. administration of αCD47-G1; and Group 4, OV-αCD47-G1. After tumor implantation, Groups 2, 3 and 4 received intracranial injection of oHSV (OV-Q1 or OV-αCD47-G1) at a dose of 2 × 10^5^ PFU per mouse on day 21. Group 1 received saline as control. On day 22, Group 3 received i.p. injection of purified αCD47-G1 at the dose of 150 µg per mouse. Groups 1, 2, and 4 received i.p. injection of saline as control. All mice were euthanized on day 23 for blood and brain harvesting. **b** The concentration of αCD47-G1 in plasma measured by ELISA in mice from different treatments. One-way ANOVA with *P* values corrected for multiple comparisons by the Bonferroni test (*n* = 3 animals). **c**, **d** Slides from the brain tissues isolated from experimental mice were subjected to H&E and immunohistochemical staining, the latter with anti-HSV, anti-human Fc, which identifies IgG, anti-CD11b or anti-NKp46 antibodies. Images with high and low magnifications are shown in (**c**) and (**d**), respectively. The boxed images in (**d**) are shown in higher power in (**c**). Data presented are representative of one (**c**, **d**) or three (**b**) mice of at least three mice in total with similar data. Data in (**b**) are presented as mean values ± SD.

To evaluate the efficacy of delivering αCD47-G1 antibody to the GBM microenvironment by i.c. OV-αCD47-G1 versus systemic intraperitoneal (i.p.) antibody administration, we modified a previously described xenograft GBM mouse model by i.c. injecting 1 × 10^5^ GBM43 cells into athymic nude mice^34^ (Fig. 4a). GBM43 tumor cells were implanted on day 0. Mice were divided into four groups for i.c. administration on day 21: Group 1, saline; Group 2, OV-Q1; Group 3, a combination of OV-Q1 plus i.p. delivery of αCD47-G1; and Group 4, OV-αCD47-G1. The viral dose for the two groups receiving an i.c. injection of OV-Q1 and the group receiving i.c. injection of OV-αCD47-G1 on day 21 was 2 × 10^5^ plaque-forming unit (PFU) per mouse. Group 3 received i.p. αCD47-G1 on day 22. Mice in other groups received i.p. saline on day 22 as control. All mice were sacrificed on day 23 to determine the levels of the antibody in plasma by ELISA and in the central nervous system (CNS) by IHC (Fig. 4a).

Mice with i.p. injection of αCD47-G1 (150 µg/mouse) had approximately 10 µg/ml of αCD47-G1 detected in the plasma, while groups 1, 2, and 4 had no or little αCD47-G1 detected in the plasma (Fig. 4b). Brains isolated from the experimental mice were used for histologic study. H&E staining was performed to distinguish GBM tissues from normal brain tissues (Fig. 4c, d). The immunohistochemical staining with an anti-HSV antibody, anti-human Fc antibody, anti-CD11b or anti-NKp46 antibody indicated oHSV-infected areas, presence of αCD47-G1, distribution of macrophages or NK cells, respectively. The anti-human Fc staining showed that there was a measurable amount of αCD47-G1 detectable in the brains of the mice i.c. treated with OV-αCD47-G1 but not in the brains of those i.c. treated with saline or OV-Q1 nor in the brains of mice that received i.c. OV-Q1 plus i.p. αCD47-G1 (Fig. 4c, d; anti-human Fc). The anti-human Fc staining localized in the same area of the anti-HSV staining in the brains treated with OV-αCD47-G1 (Fig. 4c, d; anti-HSV; anti-human Fc). CD11b(+) macrophage and NKp46(+) NK cell infiltration existed only in the tumor-bearing hemispheres but not the nontumor hemispheres (Fig. 4d; anti-CD11b; anti-NKp46). The macrophages and NK cells in the brains of saline-treated mice were mainly located at the surrounding area of the entire tumor but rarely infiltrated into the central area of the tumor; however, treatment with OV-Q1 or OV-αCD47-G1 substantially increased intratumoral infiltration of CD11b(+) macrophages and NKp46(+) NK cells (Fig. 4c, d; anti-CD11b; anti-NKp46).

To confirm our findings, we repeated the above experiment with some modifications: performing i.p. administration of αCD47-G1 or saline twice (one on day 22 and the other on day 24) and sacrificing the mice on day 25 (Supplementary Fig. 9a). Similar results were observed. Plasma αCD47-G1 (approximately 10 µg/ml) was only detectable in the group with i.c. OV-Q1 plus i.p. αCD47-G1 administration (Supplementary Fig. 9b). Compared to the saline group, the enhancement of intratumoral infiltration of CD11b(+) macrophages and NKp46(+) NK cells induced by oHSV treatment was also observed 4 days post oHSV i.c. administration (Supplementary Fig. 9c, d; anti-CD11b; anti-NKp46). αCD47-G1 was detectable both 2 and 4 days post oHSV i.c. administration only in the OV-αCD47-G1-treated brains rather than the other groups (Fig. 4c, d and Supplementary Fig. 9c, d; anti-human Fc), suggesting the continuous release of αCD47-G1 from the OV-αCD47-G1-treated GBM microenvironment.

### OV-αCD47-G1 is superior to OV-αCD47-G4 to improve oncolytic virotherapy in a xenograft GBM model

**Fig. 5:**
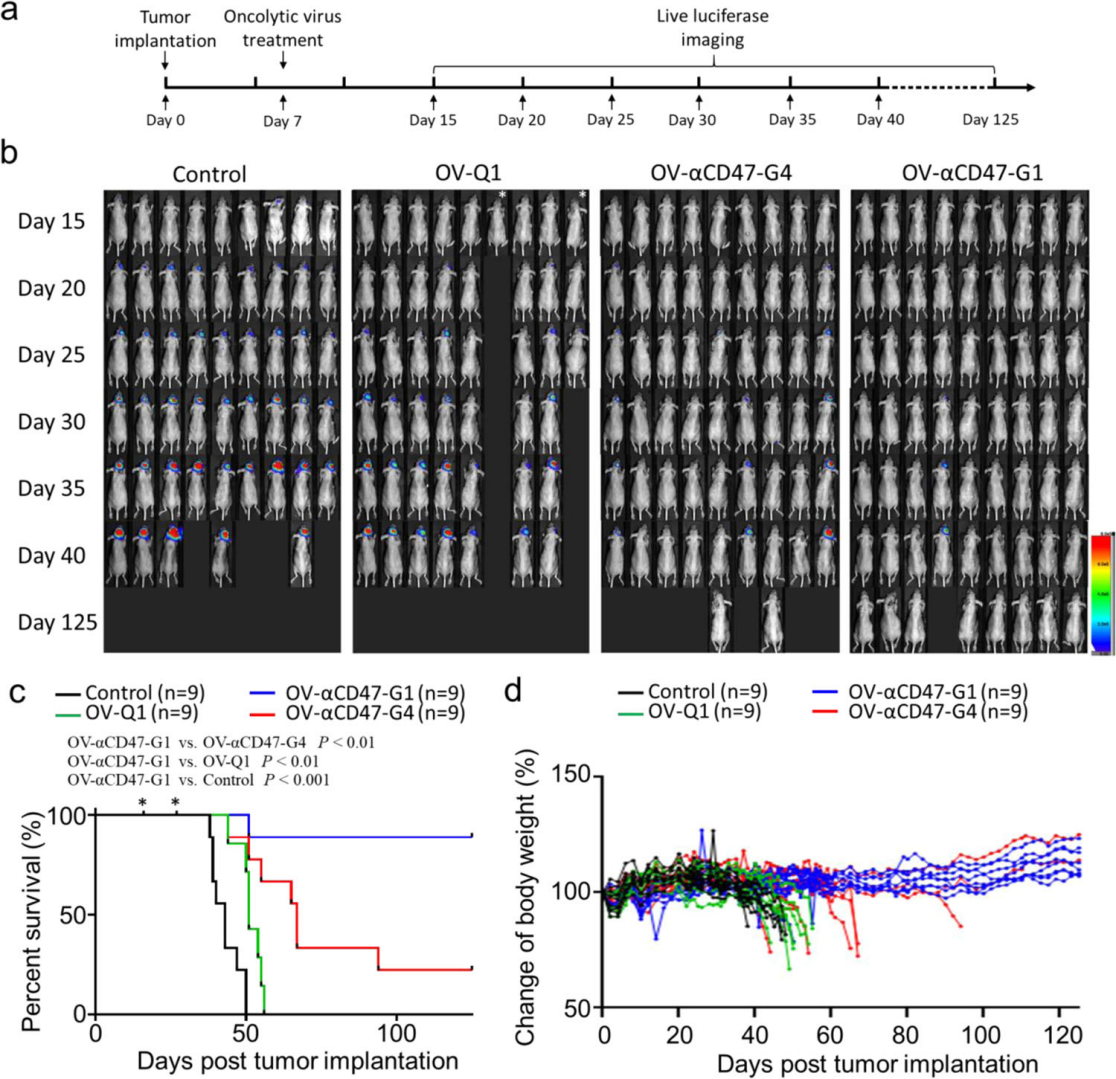
Comparison of the effectiveness of OV-αCD47-G1 versus OV-αCD47-G4 to improve in vivo oncolytic virotherapy of GBM in an orthotopic model of human GBM. **a** Experimental timeline for in vivo studies. An orthotopic model of human GBM was established by i.c. injection of 1 × 10^5^ GBM43-FFL human GBM cells into athymic nude mice. Seven days later, mice were intratumorally injected with vehicle control or 2 × 10^5^ PFU of OV-Q1, OV-αCD47-G1, or OV-αCD47-G4. **b** Time-lapse luciferase imaging of GBM43-FFL growth in mice with indicated treatments. **c** Survival of GBM43-FFL tumor-bearing mice treated as described in (**a**). Survival was estimated by the Kaplan–Meier method and compared by two-side log-rank test (*n* = 9 animals). *Two mice in the OV-Q1 control group died accidentally after i.p. injection of luciferin on days 16 and 27 without obvious GBM symptoms. **d** Individual body weights of the experimental mice were recorded once every other day.

To evaluate the efficacy of OV-αCD47-G1 and OV-αCD47-G4 for the in vivo treatment of GBM, we utilized a previously described orthotopic model of human GBM by i.c. injecting 1 × 10^5^ firefly luciferase (FFL) gene-expressing human GBM cells (GBM43-FFL) into athymic nude mice^34^. Seven days after tumor implantation, animals received an i.c. injection with OV-αCD47-G1, OV-αCD47-G4 or OV-Q1 at the dose of 2 × 10^5^ PFU per mouse, or saline as a placebo control. Tumor progression was monitored by luciferase-based imaging starting on day 15 post tumor implantation (Fig. 5a). OV-αCD47-G1 was significantly more effective than OV-αCD47-G4 at inhibiting the progression of GBM tumors in vivo, and both were superior to OV-Q1 (Fig. 5b−d). OV-Q1 slowed GBM progression moderately as compared to vehicle control and prolonged the median survival from 43 to 51 days. OV-αCD47-G4 treatment prolonged the median survival time of the GBM mice compared to both saline control and OV-Q1 treatment. Eight out of nine mice from the OV-αCD47-G1 group and two out of nine mice from the OV-αCD47-G4 group survived over 125 days without GBM symptoms and exhibited no detectable luciferase signal (Fig. 5b, c and Supplementary Fig. 10). We repeated the mouse survival experiment and found similar data (Supplementary Fig. 11). Body weight recording of the experimental mice further indicated that compared to the other groups, the mice treated with OV-αCD47-G1 were healthier with continuously increasing body weights at the late stage of the study (Fig. 5d). To evaluate the therapeutic effect of OV-αCD47-G1 on treating established GBM with bigger tumors, we repeated the survival study by delaying treatment time to 14 or 21 days vs. 7 days in Fig. 5 after tumor implantation. The results showed that mice with OV-αCD47-G1 treatment on day 14 but not on day 21 significantly prolonged survival time when compared to OV-Q1 and the saline control group (Supplementary Fig. 12).

### OV-αCD47-G1 is superior to OV-αCD47-G4 and to separate intracranial delivery of OV-Q1 and αCD47-G1 in improving outcome in an immunocompetent GBM model

**Fig. 6:**
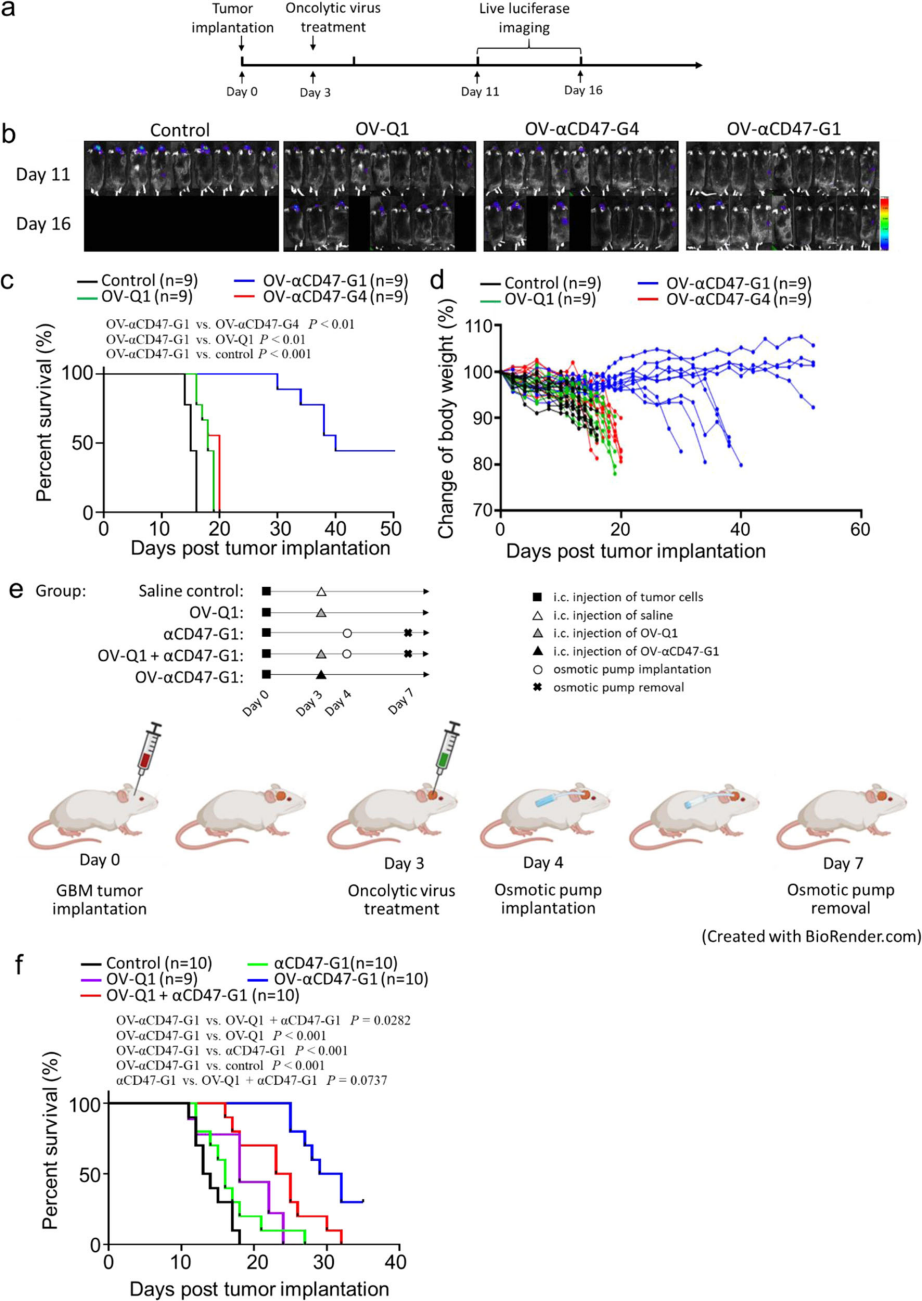
Comparison of the effectiveness of OV-αCD47-G1 versus OV-αCD47-G4 as well as combined locoregional deliveries of OV-Q1 and αCD47-G1 in an immunocompetent mouse GBM model. **a** Experimental timeline for in vivo studies. An immunocompetent mouse GBM model was established by i.c. injection of 1 × 10^5^ CT2A-hCD47 mouse GBM cells (expressing human CD47) into C57BL/6 mice. Three days later, mice were intratumorally injected with saline vehicle control or 2 × 10^5^ PFU of OV-Q1, OV-αCD47-G1 or OV-αCD47-G4. **b** Luciferase imaging of CT2A-hCD47 GBM mice with indicated treatments was taken 11 and 16 days post tumor implantation. **c** Survival of CT2A-hCD47 tumor-bearing mice as described in (**a**). Survival was estimated by the Kaplan–Meier method and compared by two-side log-rank test (*n* = 9 animals). **d** Individual body weights of the experimental mice were recorded once every other day. **e** Experimental timeline for the survival study. An immunocompetent mouse GBM model was established by i.c. injection of 1 × 10^5^ CT2A-hCD47 mouse GBM cells (expressing human CD47) into C57BL/6 mice. Three days later, mice were intratumorally injected with vehicle control or 2 × 10^5^ PFU of OV-Q1, or OV-αCD47-G1. Osmotic pumps were implanted on day 4 to the mice for continuous administration of αCD47-G1 until day 7. Mice were monitored twice a day to evaluate for tumor development. **f** Survival of CT2A-hCD47 tumor-bearing mice treated with OV-Q1, αCD47-G1, OV-Q1 plus αCD47-G1, OV-αCD47-G1, or vehicle control. Survival was estimated by the Kaplan–Meier method and compared by two-side log-rank test (*n* = 9 or 10 animals per group).

We next sought to evaluate the efficacy of OV-αCD47-G1 in an immunocompetent GBM mouse model. A modified CT2A mouse GBM model, which was susceptible to oHSV infection, was used for this purpose (Supplementary Fig. 13). The mouse CT2A GBM cells were modified to express human CD47 for generating the CT2A-hCD47 cells. The purpose for this modification was to allow the αCD47-G1 antibody to bind to the human CD47 expressed on CT2A-hCD47 GBM cells. By i.c. injection of CT2A-hCD47 cells into immunocompetent wild-type C57BL/6 mice, we established a GBM immunocompetent mouse model and repeated the survival study in Fig. 5 with slight modifications (Fig. 6a). The treatment of these mice with OV-αCD47-G1 significantly prolonged their median survival when compared to those mice treated with OV-αCD47-G4, OV-Q1, or vehicle control (Fig. 6b, c). However, in contrast to the xenograft model, we did not see a significant difference from the mice treated with OV-αCD47-G4 and either OV-Q1 or vehicle control (Fig. 6b, c). Body weight recording of the mice in the study supported the findings that OV-αCD47-G1 was the only effective treatment for the immunocompetent GBM mouse model (Fig. 6d).

Next, we repeated the survival study with the CT2A-hCD47 GBM mouse model to compare the effectiveness of i.c. administration of OV-αCD47-G1 against the combination of i.c. administration of OV-Q1 combined with continuous release of αCD47-G1 mAb into the GBM environment by an osmotic pump. For this purpose, we set up five different treatment groups of i.c. administration: Group 1, saline; Group 2, OV-Q1; Group 3, osmotic pump delivery of αCD47-G1; Group 4, a combination of OV-Q1 plus osmotic pump delivery of αCD47-G1 mAb and Group 5, OV-αCD47-G1. CT2A-hCD47 GBM cells were implanted on day 0, and 3 days later, the two groups with OV-Q1 injection (groups 2 and 4) and the group with OV-αCD47-G1 injection (group 5) received 2 × 10^5^ PFU corresponding virus per mouse. On days 4−7, the two groups with an osmotic pump delivery of αCD47-G1 received the i.c. delivery of the antibody by the pump at the rate of 1.0 µg per hour for 72 h, i.e. 24 µg per day, which is about 2−3 times of the amount produced by the injected virus, per calculation based on the data from Fig. 1f and the dose of injected virus. Mice in other groups received i.c. delivery of saline by the pump as control (Fig. 6e). As a single agent, compared to saline, both OV-Q1 and αCD47-G1 delivery by the pump showed a modest but not statistically significant improvement in mouse survival (Fig. 6f). The combination of OV-Q1 and αCD47-G1 delivery by the pump seems to be better than αCD47-G1 alone, and the *P* value is on the border of significance (*P* = 0.0737) but was not significantly different when compared to OV-Q1 alone. Consistent with the other two animal models (Figs. 5c and 6c), OV-αCD47-G1 significantly prolonged the survival of the GBM mice when compared to OV-Q1 (Fig. 6f). Importantly, OV-αCD47-G1 is the best among all the tested groups and in particular is statistically superior to the combination of i.c. OV-Q1 and αCD47-G1 delivery by the pump. As the OV-αCD47-G1 therapeutic combines OV-Q1 and αCD47-G1 into a single agent, and the single agent shows therapeutic survival outcomes superior to αCD47-G1 alone, OV-Q1 alone, and their combination, the two-in-one single agent, i.e., an oncolytic virus expressing a full-length IgG1 anti-CD47 antibody is an innovative, convenient, and effective approach for the treatment of experimental GBM.

### OV-A4-IgG2b is superior to OV-A4-IgG3 in improving outcome in a fully immunocompetent GBM model without adverse effects on red blood cells

**Fig. 7:**
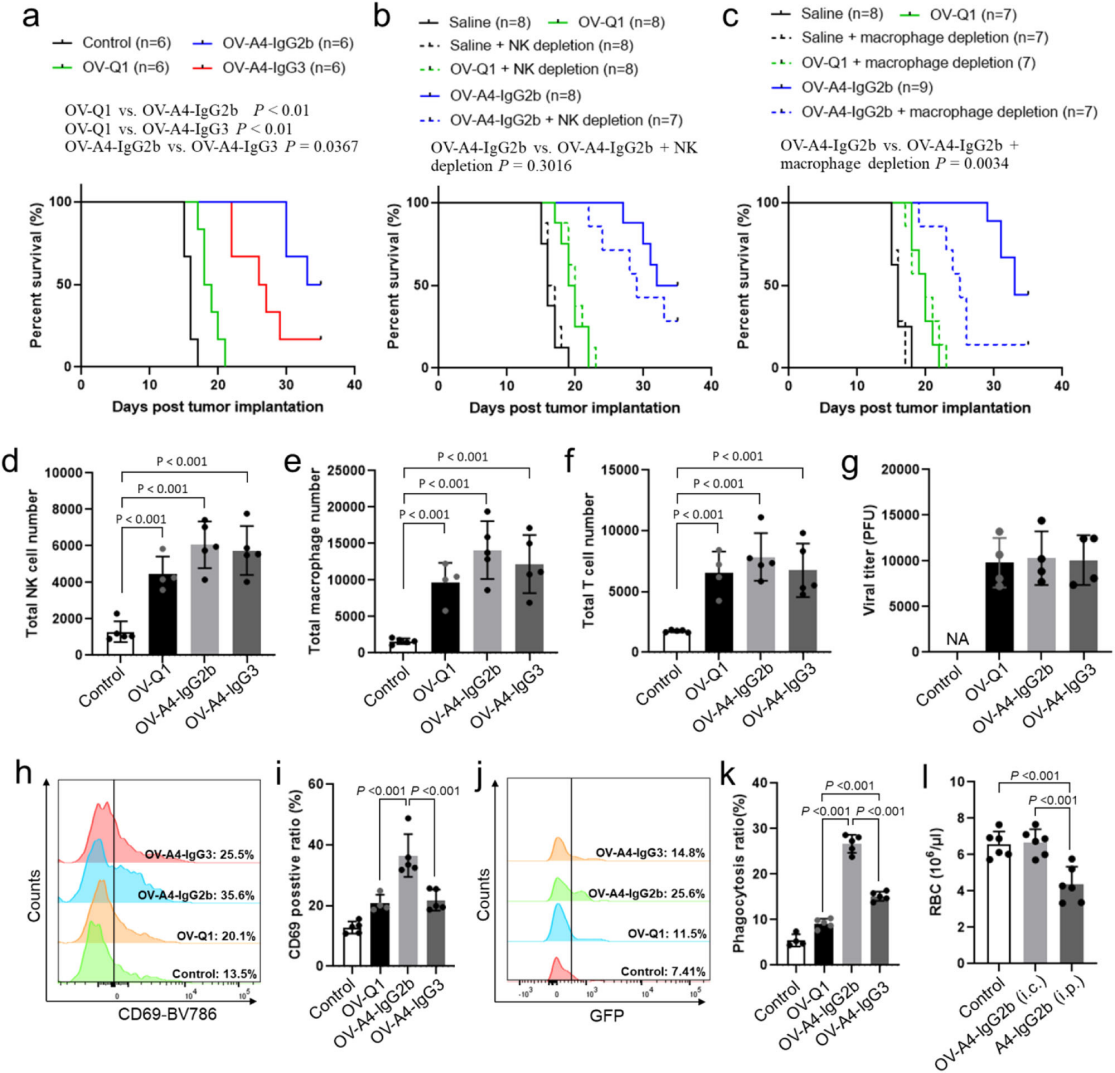
Comparison of the effectiveness of OV-A4-IgG2b versus OV- A4-IgG3 in a fully immunocompetent mouse GBM model. **a** Survival of CT2A tumor-bearing C57BL/6 immunocompetent mice treated with vehicle control, OV-Q1, OV-A4-IgG2b, or OV-4-IgG3. **b** Survival of CT2A tumor-bearing C57BL/6 immunocompetent mice treated with vehicle control, OV-Q1, or OV-A4-IgG2b in the presence or absence of NK cell depletion. **c** Survival of CT2A tumor-bearing C57BL/6 immunocompetent mice treated with vehicle control, OV-Q1, or OV-A4-IgG2b in the presence or absence of macrophage depletion. Survival was estimated by the Kaplan–Meier method and compared by two-side log-rank test. **d**−**f** Intracranial infiltration of NK cells (CD3−NKp46+) (**d**), macrophages (F4/80+CD45highCD11b+) (**e**), and T cells (CD3+NKp46−) (**f**) in CT2A-bearing mice measured by flow cytometry 3 days after virus injection (*n* = 5 animals). **g** Viral production of OV-Q1-, OV-A4-IgG2b-, and OV-A4-IgG3-treated mice. **h** CD69 expression in NK cells isolated from mouse brains 3 days after virus injection. Representative flow cytometric plots of each group (*n* = 5 animals). **i** Quantification of CD69 expression in NK cells in (**h**). **j** In vivo macrophage phagocytosis of GBM-expressing CT2A cells after oHSV treatment. Representative flow cytometric plots of each group (*n* = 6 animals). **k** Quantification of macrophage phagocytosis of GBM-expressing CT2A cells in (**j**). **l** Red blood cell counts in peripheral blood after i.c. injection of OV-A4-IgG2b or i.p. injection of purified A4-IgG2b (*n* = 6 mice). For (**d**−**g**), (**i**) and (**k**, **l**), one-way ANOVA with *P* values corrected for multiple comparisons by the Bonferroni test. Data are presented as mean values ± SD.

To avoid the cross-species interaction of human CD47 and mouse SIRPα, which is artificial, we established a fully immunocompetent mouse model by replacing the anti-human CD47 antibody with anti-mouse CD47 antibody. Briefly, we constructed the anti-mouse CD47 antibodies (Clone: A4)^35^ on mouse IgG2b and mouse IgG3 scaffolds, termed as A4-IgG2b and A4-IgG3, respectively, as well as the corresponding oHSVs expressing A4-IgG2b (OV-A4-IgG2b) or A4-IgG3 (OV-A4-IgG3). The mouse CD47 blocking effect of A4-IgG2b was validated by flow cytometry assay (Supplementary Fig. 14). The macrophage phagocytosis activating effects of A4-IgG2b and A4-IgG3 against mouse GBM cell line CT2A cells were measured. Consistent with our previous finding, A4-IgG2b, which is similar to αCD47-G1 that has a stronger Fc receptor binding affinity^36–39^, induced a significantly higher level of macrophage phagocytosis against GBM cells compared to A4-IgG3, which is similar to αCD47-G4 nearly lacking Fc receptor binding ability^36–39^ (Supplementary Fig. 15a, b). Mouse NK cell activating effect of A4-IgG2b was confirmed. Results showed that A4-IgG2b but not A4-IgG3 enhanced the ADCC of mouse NK cells against CT2A cells (Supplementary Fig. 15c). C57BL/6 mice i.c. injected with CT2A cells were used for the survival study. Three days after tumor implantation, mice were intratumorally injected with OV-Q1, OV-A4-IgG2b, OV-A4-IgG3, or vehicle control. The results showed that OV-A4-IgG2b and OV-A4-IgG3 treatments both significantly prolonged the median survival of mice when compared to the treatment of OV-Q1 or control. OV-A4-IgG2b is superior to OV-A4-IgG3 to improve oncolytic virotherapy in the fully immunocompetent GBM mouse model (Fig. 7a). To define the immunological mechanisms involved in the advanced therapeutic effect of OV-A4-IgG2b, we repeated the survival study with macrophage or NK cell depletion. The results showed that NK cell depletion slightly shortened the survival of OV-A4-IgG2b-treated mice. The median survival is 29 days for the OV-A4-IgG2b treatment group with NK cell depletion, compared to 32 days for the group without NK cell depletion (Fig. 7b). Macrophage depletion significantly shortened the survival of OV-A4-IgG2b-treated mice when compared to the group without macrophage depletion (Fig. 7c). These data indicated that macrophages, most likely NK cells as well, play roles in contributing to the effect of improving survival of GBM mice by OV-A4-IgG2b. We also performed a flow cytometry assay to study the immune cell recruitment and activation. The results showed that compared to control treatment, OV-Q1, OV-A4-IgG2b, and OV-A4-IgG3 treatments all significantly enhanced the intracranial infiltration of macrophages, NK cells, and T cells (Fig. 7d−f). There was no significant difference in both immune cell infiltration and viral production among the OV-Q1, OV-A4-IgG2b, and OV-A4-IgG3 groups (Fig. 7g). We also measured NK cell activation in vivo and found that all three oHSV treatments significantly increased the CD69 expression on NK cells. Compared to OV-Q1, OV-A4-IgG2b but not OV-A4-IgG3 showed significant stronger NK cell activating effect (Fig. 7h, i). The in vivo phagocytosis analysis of macrophages showed that OV-A4-IgG2b was significantly superior to OV-A4-IgG3, while both had significantly higher levels of phagocytosis than OV-Q1 (Fig. 7j, k). Collectively, the data from the fully immunocompetent model treated with oHSV expressing the murine version of anti-CD47 antibody backs up our data of oHSV expressing an anti-human CD47 antibody, suggesting that an oHSV expressing a full-length antibody with a strong Fc-dependent effect can effectively treat GBM with a local administration.

CD47, a crucial marker of red blood cells (RBCs), prevents the RBC elimination by macrophages^40^. The anti-CD47 murine A4 antibody was previously reported to decrease RBCs in the circulating system^41^. To investigate whether OV-A4-IgG2b adversely acted on RBCs, likely via promoting hemagglutination or RBC phagocytosis, we also performed the anemia assay with the fully immunocompetent GBM mouse model. Briefly, CT2A tumor-bearing GBM mice were intratumorally injected with 2 × 10^5^ PFU of OV-A4-IgG2b or i.p. injected with 150 μg purified A4-IgG2b 3 days after tumor implantation. Twenty-four hours later the peripheral blood of the mice was collected and the RBCs were counted. The results showed that i.p. injection of A4-IgG2b but not intratumoral injection of OV-A4-IgG2b significantly decreased the RBC counts, which indicated that i.c. administration of an oHSV expressing a full-length antibody with a strong FcR effect to treat GBM had no substantial adverse effects on RBCs (Fig. 7l).

## Discussion

In this study, we combined oncolytic virotherapy and antibody therapy into a single therapeutic agent, aiming to destroy tumor cells directly by oncolysis and indirectly by converting the “cold” immune-evasive tumor microenvironment (TME) to a “hot” TME. We developed a platform by using oncolytic virus as both a therapeutic itself and locoregional delivery vehicle to encode and deliver a full-length mAb. This two-in-one, economical, and effective reagent should be able to be administered intratumorally pre-, intra- or post-operatively. Experimentally, the local delivery of OV-αCD47-G1 leads to (1) direct tumor lysis by oHSV; (2) innate immune cell infiltration and activation at the TME by oHSV; (3) blockade of the “don’t eat me” signal normally mediated by the interaction between SIRPα and CD47 expressed by macrophages and GBM cells, respectively; (4) αCD47-G1-mediated ADCP via bridging Fcγ receptors on macrophages and CD47 on GBM cells; and (5) αCD47-G1-mediated ADCC by NK cells. These multifaceted oncolysis and immunomodulatory roles collectively halt the spread of the GBM.

The systemic administration of the IgG4 anti-CD47 antibody (αCD47-G4) has shown significant antitumor activity in several types of malignancies in both preclinical and clinical studies^18,19,21^. αCD47-G4 and αCD47-G1 should have a near-identical ability to interrupt the “don’t eat me” signal mediated by the CD47−SIRPα interaction by grafting the same complementarity determining regions (CDRs). αCD47-G4 was constructed on a human IgG4 scaffold to minimize adverse effects resulting from Fc-dependent effector functions resulting from systemic administration of αCD47-G1 such as ADCC by NK cells and ADCP by macrophages. We therefore generated OV-αCD47-G1 as well as OV-αCD47-G4, which release full-length αCD47-G1 and αCD47-G4 mAbs, respectively, to understand the importance of the Fc−FcR interaction in tumor clearance. Our results showed that the antitumor efficacy of OV-αCD47-G1 is significantly higher than that of OV-αCD47-G4 in vitro and in vivo. However, based on the results collected from the CT2A-hCD47/C57BL/6 immunocompetent GBM model, OV-αCD47-G4 did not show a significant difference when compared to OV-Q1. This model is based on CT2A-hCD47 established by overexpressing human CD47 on mouse GBM CT2A cells. Possible explanations for this include: (1) the murine macrophages still receive the murine-CD47-mediated “don’t eat me” signal from the CT2A-hCD47 GBM cells that cannot be blocked by anti-human CD47 antibodies (αCD47-G1 or αCD47-G4). Of note, the binding affinity between C57BL/6 SIRPα and murine CD47 is similar to that between C57BL/6 SIRPα and human CD47^26,42^; (2) human αCD47-G1 can still bind to a murine Fc receptor to induce ADCP or ADCC but αCD47-G4 cannot, as shown for other antibodies^43^. These suggest that in this CT2A-hCD47/C57BL/6 immunocompetent GBM model, αCD47-G4 may not completely block the “don’t eat me” signal and also lacks the Fc-dependent effect so that we did not observe the difference between OV-αCD47-G4 and OV-Q1.

Therefore, to avoid the cross-species interaction of human CD47 and mouse SIRPα, we generated OVs expressing an anti-mouse CD47 antibody, OV-A4-IgG2b and OV-A4-IgG3 to validate the OV-αCD47-G1 and -G4 results. Mouse IgG2b and IgG3 are similar to human IgG1 and IgG4, respectively, in terms of their binding affinity to Fc receptors. Similar to the OV-αCD47-G1 and -G4 results, OV-A4-IgG2b treatment showed the strongest therapeutic effect on GBM among other OV treatments or saline. Importantly, in this fully immunocompetent model, OV-A4-IgG3 also significantly prolongs the survival of experimental mice when compared to OV-Q1 without expressing an antibody but is less effective than OV-A4-IgG2b in treating GBM. These validated the data from the GBM43 xenograft model.

The above fully immunocompetent model enables us to investigate the role of immune cells mediating an oncolytic virus expressing a full-length antibody with or without the Fc-dependent effect. The immunosuppression TME of GBM, characterized by low activated T-cell infiltration and high ratio of TAMs, leads to tumor immune evasion^44^. TAMs have been found to be the major immune cells that promote tumor development in the GBM microenvironment^45,46^. Since modulating TAMs is considered to be a promising antitumor strategy, targeting the CD47-SIRPα axis of TAMs should be a good approach for the treatment of GBM. CD47 is highly expressed on numerous types of tumors, including GBM^17,28,47^. CD47 acts as an anti-phagocytic “don’t eat me” signal by binding to SIRPα on the surfaces of macrophages. Activation of SIRPα results in the inhibition of phagocytosis^19^. Expression of CD47 helps the tumor cells escape from elimination by macrophages and facilitates their metastasis^48^. Disrupting the CD47-SIRPα axis using mAbs has been shown to increase the phagocytosis against cancer cells and inhibit the progression of some hematological malignancies as well as solid tumors^18,21,49,50^. Our results showed that both OV-A4-IgG2b and OV-A4-IgG3 increased the intracranial infiltration of macrophages and phagocytosis against GBM cells in vivo and in vitro. Macrophage depletion restrained OV-A4-IgG2b virotherapy efficacy against GBM. These data indicate that macrophages play a strong effect on mediating our oHSV expressing an antibody, especially the one with the Fc-dependent effect. Moreover, we also found oHSV treatments dramatically increased the intracranial infiltration of NK cells and T cells. Collectively, our data suggest that oHSV expressing an antibody can induce both innate and adaptive immune responses to GBM.

MAbs have been widely and successfully used as targeted therapies in many cancers. However, the intravenous (i.v.), subcutaneous (s.c.), or intramuscular (i.m.) routes of administration may be impeding their efficacy in solid tumors with challenging TMEs. Because of large molecular sizes, s.c. and i.m. administration of mAbs require an additional absorption step, whereby mAbs are transported from the interstitial space into the lymphatic system prior to draining into the blood stream^51^. However, regardless of the route of administration, the systemic delivery of mAbs is mainly distributed in plasma rather than the targeted tissue. To achieve an effective local dose in targeted tissues such as solid tumors, a high systemic dose of mAb is required but this can result in adverse side effects and excessive cost. Moreover, traversing the BBB for mAb treatment of GBM is another challenge that largely precludes the systemic approach for mAb therapy in most GBM patients. Our antibody distribution data comparing systemic to locoregional delivery of the αCD47-G1 molecule suggest that the locoregional delivery of αCD47-G1 to the TME by OV-αCD47-G1 is an effective approach, even when compared to delivering the combination of oHSV and αCD47-G1 locoregionally but delivering them as two separate therapeutics.

The current study suggests that re-engineered oHSV is an excellent platform to deliver mAbs in solid tumors such as GBM because the mAbs can be delivered beyond the BBB locoregionally by the GBM itself given the intratumorally administrated oHSV. In our current approach, the full-length antibodies, including both heavy and light chains produced by a strong viral promoter, can increase the locoregional delivery of antibodies in the TME but eliminate toxicities and would be required to achieve superior therapeutic levels in the CNS. However, when systemic delivery of oncolytic virus is considered for tumors outside of the CNS, OV-αCD47-G4 may prove to be effective with less toxicity. Considering that both the oHSV backbone and anti-CD47 antibody have been tested in the clinic, our oHSV expressing an anti-CD47 antibody should likely be considered for translation into the clinic for the treatment of systemic solid tumors. Current good manufacturing practices manufacturing of OV-αCD47-G1 has been completed at our institution.

Our therapeutic oHSV will not only activate the innate immune system but also continuously produce the mAb locally as long as the OV persists within the TME. In the ideal situation, once tumor cells are completely lysed by the OV, replicating virus dissipates and antibody production will cease, thereby limiting the potential for long-term local tissue toxicity. Although our current approach is designed to produce a full-length antibody to directly target immune cells with IgG1 and IgG4 options, our platform can be used to deliver other forms of mAbs such as single-chain mAbs (scFv) or any mAb with diverse functions such as T-cell checkpoint blockade including anti-PD1, anti-PD-L1, anti-CTL4 or mAbs that directly target tumor cells, e.g., the anti-EGFR mAb^52,53^.

In summary, we have developed an effective oHSV platform that provides locoregional delivery of full-length mAbs to treat GBM by combining direct tumor lysis, innate immune infiltration and activation, immune checkpoint inhibition of macrophages, and Fc-dependent innate immune cell cytotoxic functions. Our platform can be extended to express other transgenes to target immune cells and/or tumor cells in the TME and therefore has the potential to enhance the overall efficacy of both oncolytic virotherapy and mAb therapy for the treatment of cancer,

## Methods

### Ethics statement

Experiments and handling of mice were conducted under federal, state, and local guidelines and with an approval from the City of Hope Animal Care and Use Committee. To isolate human monocytes and NK cells, peripheral blood cones were collected from healthy donors after written informed consent under a protocol approved by the City of Hope Institutional Review Board.

### Cells

Gli36ΔEGFR, U251T2, LN229, GBM30, Vero, and CT2A cells were obtained from the laboratory of E. Antonio Chiocca. GBM43 and BT422 were developed at Mayo Clinic. The original commercial source of Vero cells, U251T2 and LN229 is from ATCC. CT2A cells were originally purchased from MilliporeSigma.

Human GBM cell lines (Gli36ΔEGFR, U251T2, and LN229) and mouse GBM cells (CT2A and CT2A-hCD47) were cultured with DMEM supplemented with 10% FBS, penicillin (100 U/ml), and streptomycin (100 μg/ml). CT2A-hCD47 cells were generated by transfecting the murine CT2A cell line to express the human CD47 gene. GBM43 spheroid cells derived from a GBM patient and modified to express an FFL gene were named GBM43-FFL and used for in vivo imaging. GBM43 cells with CD47-knockout, termed as GBM43ΔCD47, were constructed by the CRISPR/Cas9 system. GBM43, GBM43-FFL cells, and GBM43ΔCD47 were maintained as tumor spheres with basic neurobasal medium supplemented with 2% B27 (Gibco), human epidermal growth factor (EGF, 20 ng/ml), and fibroblast growth factor (FGF, 20 ng/ml) in low-attachment cell culture flasks. BT422 cells derived from a GBM patient were cultured with NS-A basal medium (StemCell) with EGF (20 ng/ml) and FGF (20 ng/ml) in low-attachment cell culture flasks. Monkey kidney epithelium-derived Vero cells used for viral propagation and plaque-assay-based viral titration were maintained with DMEM supplemented with 10% FBS, penicillin (100 U/ml), and streptomycin (100 μg/ml).

### Production of αCD47-G1, αCD47-G4, A4-IgG2b, and A4-IgG3

CHO cells were used to produce αCD47-G1, αCD47-G4, A4-IgG2b, and A4-IgG3 for functionality tests. For αCD47-G1 and αCD47-G4, the light-chain and heavy-chain coding genes of αCD47-G4 were reconstructed into the lentivirus system^22^. αCD47-G1 used the same light-chain coding gene as αCD47-G4, but a modified αCD47-G4 heavy-chain coding gene that replaced the human IgG4 constant region with the human IgG1 constant region as the heavy-chain coding gene. For A4-IgG2b and A4-IgG3, the DNA sequences encoding anti-mouse CD47 VHH nanobody (clone A4) was fused with mouse IgG2b or IgG3, respectively. Lentiviral vectors were used to transduce CHO cells to express αCD47-G1, αCD47-G4, A4-IgG2b, or A4-IgG3. For αCD47-G1 and αCD47-G4, the light-chain and heavy-chain coding genes were carried by different lentiviral vectors with GFP and mCherry selection markers, respectively, for sorting the double-positive CHO cells using a FACS Aria II cell sorter (BD Biosciences, San Jose, CA, USA). For A4-IgG2b and A4-IgG3, the fusion protein was carried by the pCDH lentiviral vector with a GFP selection marker, which was transduced into CHO and subsequently purified using a FACS Aria II cell sorter (BD Biosciences, San Jose, CA, USA). The conditioned supernatants of the lentivirus-infected CHO cells were used to purify αCD47-G1, αCD47-G4, A4-IgG2b, and A4-IgG3by using a protein G column (Thermo Fisher, 89927). For the in vivo test, the purified αCD47-G1 and A4-IgG2b were desalted by fast protein liquid chromatography.

### CD47 binding and blocking assays

U251T2 cells pre-blocked with 2% BSA were incubated with 0, 5, 10, 25, 50,100, 250, 500, 1000, 2500, 5000, and 10,000 ng/ml αCD47-G1 or αCD47-G4 antibodies purified from transduced CHO cells for 30 min. Then the cells were washed twice and stained with APC-conjugated anti-human Fc (Jackson ImmunoResearch, 209-605-098) for 20 min. After that, the cells were washed twice and stained with BV786-conjugated anti-human CD47 antibody (clone, B6H12, BD Biosciences, 563758) for 20 min. The cells were analyzed by using Fortessa X20 flow cytometer (BD Biosciences). Median fluorescence intensity (MFI) of APC and BV786 was used to determine CD47 binding and blocking capacity of αCD47-G1 and αCD47-G4. For the blocking capacity of A4-IgG2b, similar procedures were performed with CT2A cells and purified A4-IgG2b. APC-conjugated anti-mouse CD47 antibody (clone, miap301, Biolegend, 127513) was used as a detecting antibody (Supplementary Fig. 16).

### Measurement of antibody concentration

U251T2 cells were infected with OV-Q1, OV-αCD47-G1, or OV-αCD47-G4 at a multiplicity of infection (MOI) of 2. Two hours after, the infection media were replaced with fresh media. The supernatants from each group were then harvested at 6, 12, 24, 48, and 72 hpi to measure antibody concentration by ELISA. A series amount of αCD47-G1 and αCD47-G4 antibodies purified from transduced CHO cells with known concentrations severed as standards. ELISA was performed with slight modification^54^. Briefly, the recombinant human CD47 protein (Abcam, ab174029) was used as a coating reagent. An anti-human Fc antibody (Sigma, MAB1307) was used as a detecting antibody.

### Generation of OV-αCD47-G1, OV-αCD47-G4, OV-A4-IgG2b, and OV-A4-IgG3

OV-αCD47-G1 and OV-αCD47-G4, OV-A4-IgG2b, and OV-A4-IgG3 were generated by following published protocols with some modifications^4,23^. For OV-αCD47-G1 and OV-αCD47-G4, to drive expressions of the light chain and heavy chain simultaneously with a strong viral promoter, the light-chain and heavy-chain coding genes were linked with a DNA sequence encoding a T2A self-cleaving peptide. The linked αCD47-G1 and αCD47-G4 light-chain and heavy-chain sequences were inserted into pT-oriSIE4/5 following the HSV pIE4/5 promoter to construct pT-oriSIE4/5-αCD47-G1 or pT-oriSIE4/5-αCD47-G4. The A4-IgG2b and A4-IgG3 fusion proteins were inserted into pT-oriSIE4/5 following the HSV pIE4/5 promoter to construct pT-oriSIE4/5-A4-IgG2b or pT-oriSIE4/5-A4-IgG3. pT-oriSIE4/5-αCD47-G1, pT-oriSIE4/5-αCD47-G4, pT-oriSIE4/5-A4-IgG2b, pT-oriSIE4/5-A4-IgG3, or pT-oriSIE4/5 was recombined with fHsvQuik-1 for engineering OV-αCD47-G1, OV-αCD47-G4, OV-A4-IgG2b, OV-A4-IgG3, and OV-Q1, respectively. Vero cells were used for propagating and titrating the viruses. Virus titration was performed using plaque assays. Briefly, monolayer Vero cells were seeded in a 96-well plate. After 12 h, these cells were infected with gradient-diluted viral solutions. The infection media were replaced with DMEM supplemented with 10% FBS, after a 2-h infection. GFP-positive plaques were observed and counted with a Zeiss fluorescence microscope (AXIO observer 7) 2 days after infection to calculate the viral titer. To concentrate and purify the viral particles, the culture media containing viruses were harvested and centrifugated at 3000 × *g* for 30 min. Then the supernatants were collected and ultra-centrifugated at 100,000 × *g* for 1 h. The virus pellets were resuspended with saline as needed.

### Oncolysis and viral production assay

The in vitro oncolysis assay was measured by the CCK8 kit with slight modification^55^. Briefly, U251T2 and Gli36ΔEGFR cells were seeded onto 96-well plates at densities of 5000 cells/well and allowed to attach for 24 h. Cells were then treated with graded concentrations of OV-Q1, OV-αCD47-G1, or OV-αCD47-G4. After 3 days, cell lysis was measured by using the cell counting kit-8 (CCK-8, Abcam, ab228554) following the manufacturer’s protocol. CCK8 tetrazolium salt is reduced by cellular dehydrogenases to an orange formazan product that is soluble in a tissue culture medium. The amount of formazan produced is directly proportional to the number of living cells and is measured by absorbance at 460 nm. Cell lysis and viral dose curves were established to evaluate the in vitro oncolytic efficacy of OV-αCD47-G1 and OV-αCD47-G4.

The viral replication assay was performed by plaque assay^4^. Briefly, monolayers of U251T2 and Gli36ΔEGFR cells were seeded on 96-well plates and infected with OV-Q1, OV-αCD47-G1, or OV-αCD47-G4 at an MOI of 2. Two hours after infection, the infection media were replaced with fresh media. The supernatants from each group were then harvested at 24, 48 and 72 hpi, and viral titers were determined by plaque assays.

### Isolation and culture of macrophages

For isolating and culturing mouse BMDMs, BALB/c mice were sacrificed at the time of bone marrow harvest. Bone marrow cells were extracted from the tibias and femurs by flushing with culture medium using a 25-G needle. The cells were then passed through a 70 µm nylon mesh (BD Biosciences) and washed three times with PBS. Extracted BM cells were implanted with 2.4 × 10^7^/100 mm culture dish (BD Falcon) and cultured for 7 days in the presence of murine M-CSF (PeproTech, 315-02) medium (replacing culture medium on days 3 and 5).

For isolating and culturing human primary macrophages, peripheral blood was collected from healthy donors. Human monocytes were isolated and enriched by using the RosetteSep™ Human Monocyte Enrichment Cocktail kit (Stemcell, Cat#15068) from the peripheral blood. The enriched human monocytes were cultured with RPMI-1640 medium containing 20 ng/ml human M-CSF (PeproTech, Cat#300-25-50UG) and 2% human serum for 7 days to induce the macrophage differentiation (replacing culture medium on days 3 and 5).

### Flow cytometry-based phagocytosis assay

For the phagocytosis assay of mouse BMDMs, GBM43 and BT422 cells stained with CFSE (Thermo Fisher, C34554) were used as target cells. BMDMs and target cells were cocultured at a ratio of 1:2 for 2 h in the presence of vehicle control, αCD47-G1 or αCD47-G4 at the dose of 5 μg/ml, in a humidified, 5% CO_2_ incubator at 37 °C in ultra-low-attachment 96-well U-bottom plates (Corning) in serum-free 1640 (Life Technologies). Then the cells were harvested by centrifuging at 400 × *g* for 5 min at 4 °C and stained with anti-mouse CD11b (BD Biosciences, 552850) to identify macrophages. For blocking Fc receptors, BMDMs were pre-incubated with 10 µg/ml isotype human IgG1 (Biolegend, 403505) for 30 min. For the phagocytosis assay of A4-IgG2b and A4-IgG3, CT2A cells pre-incubated with PBS vehicle control, A4-IgG2b, or A4-IgG3 at a concentration of 5 μg/ml were used as target cells, following the similar procedures mentioned above. All flow cytometry data were collected using a Fortessa X20 flow cytometer (BD Biosciences). Phagocytosis was measured as the number of CD11b+CFSE+ macrophages, quantified as a percentage of the total CD11b+ macrophages (Supplementary Fig. 16).

For the phagocytosis assay of human primary macrophages, GBM43 cells stained with CFSE (Thermo Fisher, C34554) were used as target cells. Human macrophages and target cells were cocultured at a ratio of 1:2 for 4 h in the presence of vehicle control, αCD47-G1 or αCD47-G4 at the dose of 5 μg/ml, in a humidified, 5% CO_2_ incubator at 37 °C in ultra-low-attachment 96-well U-bottom plates (Corning) in serum-free 1640 (Life Technologies). Then the cells were harvested by centrifuging at 400 × *g* for 5 min at 4 °C and stained with anti-human CD11b (BD Biosciences, 552850, 5 μl/sample) to identify macrophages. All flow cytometry data were collected using a Fortessa X20 flow cytometer (BD Biosciences) with BD FACSDiva version 6 software (BD Biosciences). Phagocytosis was measured as the number of CD11b+CFSE+ macrophages, quantified as a percentage of the total CD11b+ macrophages (Supplementary Fig. 16).

### NK cell cytotoxicity and activation assay

GBM43, BT422, U251T2, GBM30, LN229, and Gli36ΔEGFR cells were used as target cells. Primary human NK cells isolated from leukocyte cones of healthy donors using an NK cell isolation kit (MACSxpress Miltenyi Biotec, San Diego, CA) and an erythrocyte depletion kit (Miltenyi Biotec) were used as effector cells. The target cells were labeled with ^51^Cr for 1 h. GBM43, BT422, U251T2, GBM30, and LN229 cells were cocultured with 1 µg/ml αCD47-G1 or αCD47-G4 antibodies or vehicle for 30 min. Gli36ΔEGFR cells were cocultured with gradient doses of αCD47-G1 or αCD47-G4 antibodies or vehicle for 30 min. Then the target cells were cocultured with isolated human primary NK cells at different effector (E):target (T) ratios at 37 °C for 4 h. ^51^Cr release was measured with a MicroBeta^2^ microplate radiometric counters (Perkin Elmer, Waltham, MA). Target cells incubated in complete media or 1% SDS media were used for spontaneous or maximal ^51^Cr release control, respectively. The cell lysis percentages were calculated using the standard formula: 100 × (cpm experimental release – cpm spontaneous release) / (cpm maximal release – cpm spontaneous release). The assays were performed in at least three technical replicates with NK cells from different donors. The expression of CD69, an NK cell activation marker, was measured after 4-h coculture of NK cells and GBM43, GBM30 or U251T2 cells at a ratio of 1:1 with flow cytometer by using the anti-CD56 (BD Biosciences, 557919, 5 μl/sample) and anti-CD69 (BD Biosciences, 562883, 5 μl/sample) antibodies (Supplementary Fig. 16). The granzyme B expression of NK cells was measured using the anti-granzyme B antibody (BD Biosciences, 563388). The information of the antibodies that were used in this study is provided in Supplementary Table 1.

### Immunoblot and quantitative PCR

Immunoblotting was performed following a published protocol^4^. U251T2 cells were infected with OV-Q1, OV-αCD47-G1, or OV-αCD47-G4 at an MOI of 2. Two hours after infection, the infection media were replaced with fresh media. Twenty-four hours later, the supernatants (10 ml) from each group were used to extract total proteins by a chloroform-methanol method for immunoblotting of αCD47-G1 and αCD47-G4. Ten milliliters supernatants from a 24-h culture of transduced CHO cells was also used to extract total proteins by a chloroform-methanol method for immunoblotting. Anti-human IgG heavy chain (Sigma, MAB1307), anti-human kappa chain (Thermo Fisher, MA5-12117), and the corresponding secondary antibodies (LI-COR, 925-32210) were used to detect the heavy chain and light chain.

To evaluate the effect of αCD47-G1 and αCD47-G4 on activating transcription of typical mouse macrophage cytokine genes, mouse BMDMs and GBM43 were cocultured at a ratio of 1:1 for 6 h with or without the presence of 5 µg/ml αCD47-G1 or αCD47-G4. Then the total RNA was extracted for reverse transcription to make cDNA to detect relative mRNA transcription levels of murine *Arg1, Ccl2, Ccl4, Il1b, Il6, Il10, Il12b*, and *Nos2* genes with corresponding primers. 18s rRNA was used as internal control. To evaluate the effect of αCD47-G1 and αCD47-G4 on activating transcription of typical human macrophage cytokine genes, human macrophages and GBM43 were cocultured at a ratio of 1:1 for 6 h with or without the presence of 5 µg/ml αCD47-G1 or αCD47-G4. Then the total RNA was extracted for reverse transcription to make cDNA to detect relative mRNA transcription levels of human *IL1B, IL6, IL10, IL12A*, and *NOS2* genes with corresponding primers. 18s rRNA was used as internal control. The information of the primers that were used to detect relative mRNA expression levels is provided in Supplementary Table 2. Applied Biosystems StepOnePlus real-time PCR system and QuantStudio 12 K Flex software V1.2.4 were used to collect real-time PCR data.

### Animal study

All animals in this study were housed in City of Hope Animal Facility with a light cycle with a 12-light/12-dark cycle and temperatures of 65−75 °F (~18−23 °C) with 40−60% humidity. All the mice were euthanized by carbon dioxide at the endpoints of survival studies or the indicated time points of other experiments. Six- to eight-week-old female athymic nude mice (Stock Number #002019) were purchased from Jackson Laboratories (Bar Harbor, Maine). For the survival studies, mice were anesthetized and stereotactically injected with 1 × 10^5^ GBM43-FFL cells, which express a firefly luciferase (FFL) gene, into the right frontal lobe of the brain (2 mm lateral and 1 mm anterior to bregma at a depth of 3 mm). The cells grew for 7 days, and animals were subsequently randomly divided into groups that were i.c. injected either with 2 × 10^5^ PFU oHSV (OV-Q1, OV-αCD47-G1 or OV-αCD47-G4) in 3 μl of saline or saline alone as control. Mice were subsequently monitored and weighed frequently for GBM disease progression. Luciferase-based in vivo images were taken at indicated time points to evaluate the tumor development. Mice were euthanized when they became moribund, with neurologic impairments and obvious weight loss.

For the in vivo anti-CD47 antibody distribution study, nude mice were stereotactically injected with 1 × 10^5^ GBM43 into the same site of the brain mentioned above. The GBM cells were allowed to grow for 21 days, and the mice were randomly divided into four groups: Group 1, saline; Group 2, OV-Q1; Group 3, a combination of OV-Q1 plus i.p. delivery of αCD47-G, and Group 4, OV-αCD47-G1. Group 1 received saline as control. Groups 2, 3, and 4 with OV treatment were i.c. injected with 2 × 10^5^ PFU oHSV (OV-Q1 or OV-αCD47-G1) on day 21. Group 3 received i.p. injection of purified αCD47-G1 once at the dose of 150 µg per mouse on day 22, while other groups received i.p. saline as control. Mice were euthanized on day 23 for harvesting blood and brain. To assess the continuous release of anti-CD47 antibody from OV-αCD47-G1 infection, the above experiment was modified with the following changes: Group 3 received i.p. injection of purified αCD47-G1 twice at the dose of 150 µg per mouse on day 22 and 24, while other groups received i.p. saline as control. Mice were euthanized on day 25 for harvesting blood and brain. αCD47-G1 levels in the plasma of the experimental mice were measured by ELISA. Brains isolated from the experimental mice were used for histologic study.

For establishing the immunocompetent mouse GBM model, 6- to 8-week-old female C57BL/6 mice (Stock Number #000664) were purchased from Jackson Laboratories (Bar Harbor, Maine) and housed in City of Hope Animal Facility. Mice were anesthetized and stereotactically injected with 1 × 10^5^ CT2A-hCD47 cells, which express a human CD47 gene, into the right frontal lobe of the brain (2 mm lateral and 1 mm anterior to bregma at a depth of 3 mm). The cells grew for 3 days, and animals were randomly divided into groups that were i.c. injected either with 2 × 10^5^ PFU oHSV (OV-Q1, OV-αCD47-G1 or OV-αCD47-G4) in 3 μl of saline or saline alone as control. Mice were subsequently monitored and weighed frequently for GBM disease progression. Mice were monitored and used to take luciferase-based in vivo images as indicated above.

For assessment of the effect of OV-A4-IgG2b and OV-A4-IgG3 in treating GBM in a fully immunocompetent model, 6- to 8-week-old female C57BL/6 mice were anesthetized and stereotactically injected with 1 × 10^5^ CT2A cells. Three days later, animals were subsequently randomly divided into groups that were i.c. injected either with 2 × 10^5^ PFU oHSV (OV-Q1, OV-A4-IgG2b or OV-A4-IgG3) in 3 μl of saline or saline alone as control. Mice were subsequently monitored and weighed frequently for GBM disease progression.

To continuously deliver αCD47-G1 into the tumor site in the CT2A GBM mouse model, osmotic pumps (Alzet, Cat#1003D) were filled with 100 µl of desalted and concentrated αCD47-G1 at the concentration of 1 µg/µl. The pumps were connected with the brain infusion kit (Alzet, Cat#0008851) and implanted into the mice for continuous delivery of αCD47-G1 to the tumor site at a pump speed of 1 µl per hour.

Survival studies involving immune cell depletions were performed using the same immunocompetent mouse GBM model with CT2A cells and OV-A4-IgG2b mentioned above. NK cells were depleted by i.p. injections of NK1.1 neutralizing antibody (BioXcell, BE0036, clone PK136; 0.2 mg/mouse) twice, once on the day before virus injection and once 2 days after the virus injection. Macrophages were depleted by i.p. injections of clodronate liposomes (0.2 ml/mouse) using the same schedule for NK cell depletion. The corresponding isotypes and liposomes were used as controls.

The in vivo phagocytosis assay was performed using the similar immunocompetent mouse GBM model as indicated above, except that CT2A cells were modified to express GFP as a marker. Mononuclear cells in the brain were extracted with Percoll and stained with anti-CD45 (BD Biosciences, 559864, 5 μl/sample), anti-CD11b (BD Biosciences, 552850, 5 μl/sample), and anti-F4/80 (Thermo Fisher, 12-4801-82, 5 μl/sample) antibodies to identify macrophages (Supplementary Fig. 16). The percentage of GFP+ macrophages indicating phagocytosis was analyzed using the Fortessa X-20 flow cytometer.

### Immunohistochemistry assay

Brains isolated from the experimental mice were placed in 10% neutral buffered formalin for a minimum of 72 h. After paraffin embedding, 4-μm-thick sections were cut from the blocks. H&E staining and immunohistochemical staining with anti-HSV (Cell marque, 361A-15-ASR), anti-human Fc (Jackson ImmunoResearch, 109-005-098), anti-CD11b (Abcam, ab133357), and anti-NKp46 (R&D System, AF2225) antibodies were performed by the Pathology Core of Shared Resources at City of Hope Beckman Research Institute and National Medical Center. Stained slides were mounted and scanned for observation.

### Flow cytometry

Mononuclear cells in the brain were extracted with Percoll and stained with anti-NKp46 (Biolegend, 137618, 5 μl/sample), anti-CD3 (BD Biosciences, 553066, 5 μl/sample), anti-CD45 (BD Biosciences, 559864, 5 μl/sample), anti-CD11b (BD Biosciences, 552850, 5 μl/sample), anti-F4/80 (Thermo Fisher, 12-4801-82, 5 μl/sample), and anti-CD69 (BD Biosciences, 564683, 5 μl/sample) antibodies for flow cytometric analysis of immune cell brain infiltration (Supplementary Fig. 16). The flow cytometric assessments of murine immune cells were performed with at least four independent animals. All flow cytometry data were collected using the Fortessa X-20 flow cytometer.

### Statistical analysis

Descriptive statistics (means, standard deviations, median, counts, etc.) are used to summarize data. Continuous endpoints that are normally distributed with or without prior log transformation were compared between two or more independent conditions by Student’s *t* test or one-way ANOVA, respectively. For data with repeated measures from the same subject/donor, a linear mixed model was used to compare matched groups by accounting for the underlying variance and covariance structure. *P* values were adjusted for multiple comparisons by Holm’s procedure or the Bonferroni method. For survival data, survival functions were estimated by the Kaplan–Meier method and compared by log-rank test. All tests were two-sided. A *P* value of 0.05 or less was defined as statistically significant. Statistical software GraphPad, R.3.6.3. and SAS 9.4 were used for the statistical analysis.

### Reporting summary

Further information on research design is available in the Nature Research Reporting Summary linked to this article.

## Supplementary information

Supplementary Information

Reporting Summary

## Data Availability

All original data underlying selected data shown in the figures and supplemental figures are available from the corresponding authors upon reasonable request. Source data are provided with this paper.

## Source data

Source Data
