# Supplementary material for: An oncolytic virus expressing a full-length antibody enhances antitumor innate immune response to glioblastoma
Source: Nat Commun. 2021 Oct 8;12:5908. doi: 10.1038/s41467-021-26003-6 (PMC8501058; doi:10.1038/s41467-021-26003-6)
Supplement: Supplementary file 1 — Supplementary Information [file 41467_2021_26003_MOESM1_ESM.pdf]

Supplementary Information

An oncolytic virus expressing a full-length antibody enhances antitumor innate immune response to glioblastoma

Bo Xu[#1], Lei Tian[#1], Jing Chen[#2], Jing Wang[1], Rui Ma[1], Wenjuan Dong[1], Aimin Li[3], Jianying Zhang[4], E. Antonio Chiocca[5], Balveen Kaur[6], Mingye Feng[2], Michael A. Caligiuri[1,7,8*], and Jianhua Yu[1,2,7,8*]

[1]Department of Hematology and Hematopoietic Cell Transplantation, City of Hope National Medical Center, Los Angeles, California, USA.

[2]Department of Immuno-Oncology, Beckman Research Institute, City of Hope Comprehensive Cancer Centre, Los Angeles, California, USA.

[3]Pathology Core of Shared Resources Core, Beckman Research Institute, City of Hope National Medical Center, Los Angeles, California, USA.

[4]Department of Computational and Quantitative Medicine, City of Hope National Medical Center, Los Angeles, California, USA.

[5]Department of Neurosurgery, Brigham and Women's Hospital and Harvey Cushing Neuro-oncology Laboratories, Harvard Medical School, Boston, Massachusetts, USA

[6]The Vivian L. Smith Department of Neurosurgery, Mc Govern Medical School, University of Texas, University of Texas Health Science Center at Houston, Houston, Texas, USA.

[7]Comprenhensive Cancer Center, City of Hope, Los Angeles, California, USA.

[8]Hematologic Malignancies Research Institute, City of Hope National Medical Center, Los Angeles, California, USA.

# These authors equally contributed to this work.

*Corresponding authors: Michael A. Caligiuri, MD, mcaligiuri@coh.org; Jianhua Yu, PhD, jiayu@coh.org

Supplementary Figure 1

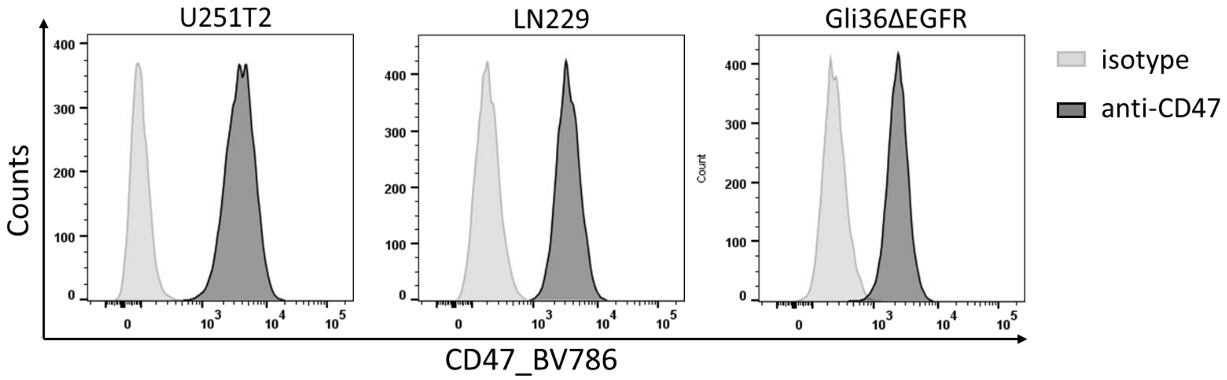

Supplementary Figure 1. CD47 expression on human GBM cells. CD47 expression on human GBM cell lines, U251T2, Gli36ΔEGFR, and LN229, were assessed by flow cytometry using a BV786-conjugaged anti-CD47 antibody.

Supplementary Figure 2

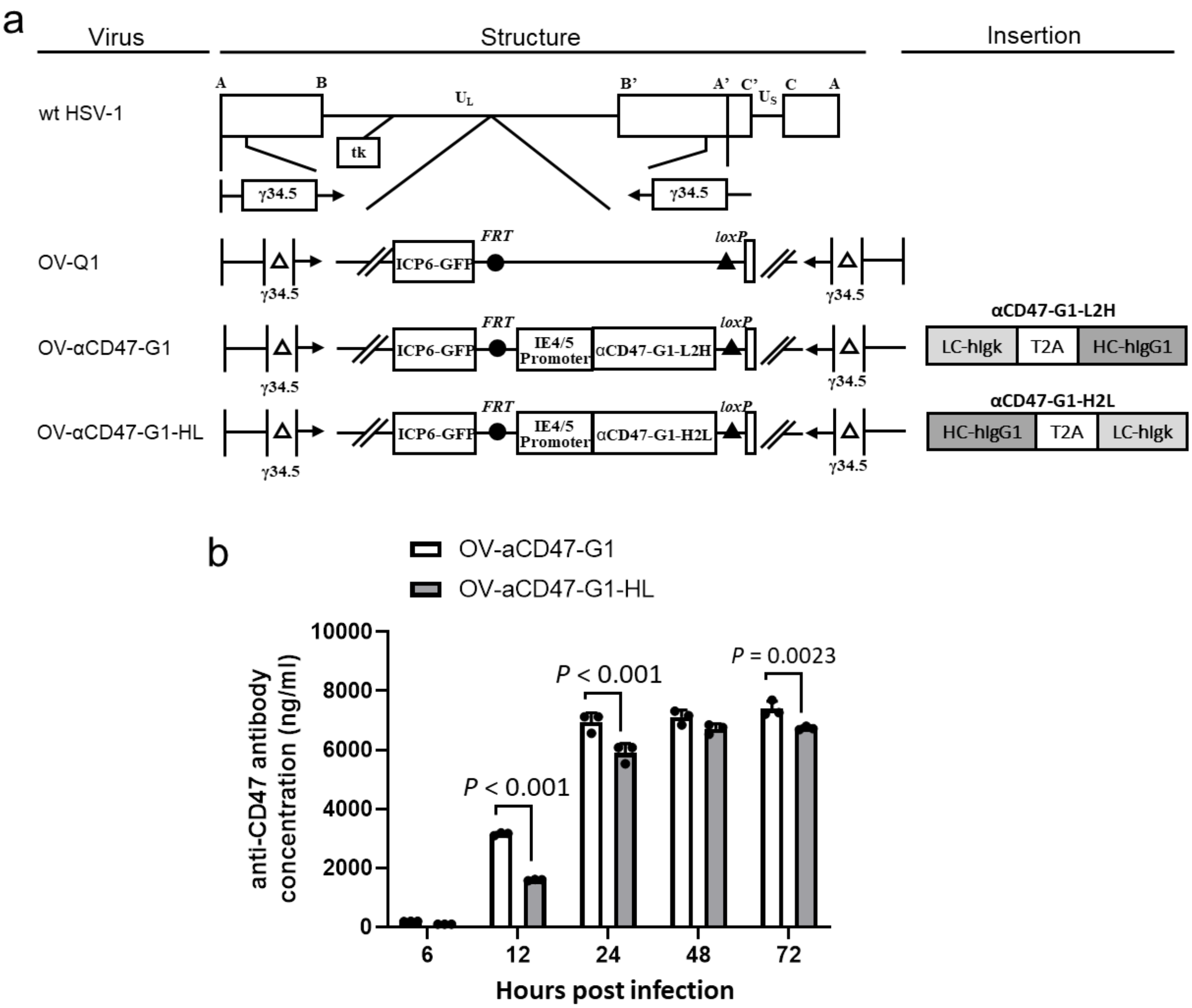

Supplementary Figure 2. Construction and characterization of OV-Q1, OV-αCD47-G1, and OV-αCD47-G1-HL. (a) Schematic of oncolytic viruses used in this study. Top: genetic map of wild type HSV-1. Second: genetic map of control oHSV, OV-Q1, with deletion of two copies of γ34.5, dysfunction of ICP6, and insertion of the GFP gene. Third: genetic map of OV-αCD47-G1 showing the inserted coding gene of IgG1 version of anti-CD47 (αCD47-G1) that is constructed on a human IgG1 scaffold. The light chain and heavy chain coding genes of αCD47-G1 linked by a T2A sequence are driven by the viral pIE4/5 promoter. Fourth: The genetic map of OV-αCD47-G1-HL showing the switched positions of light chain and heavy chain coding genes compared to OV-αCD47-G1. **(b)** αCD47-G1 yields of OV-αCD47-G1- and OV-αCD47-G1-HL-infected U251T2 cells were tested by flow cytometry assay. Differences in detection were assayed at the indicated time points. Data are representative of three independent experiments with similar data. Data are presented as mean values +/- SD (n = 3). Statistical analyses were performed by two-side Student's t test.

Supplementary Figure 3

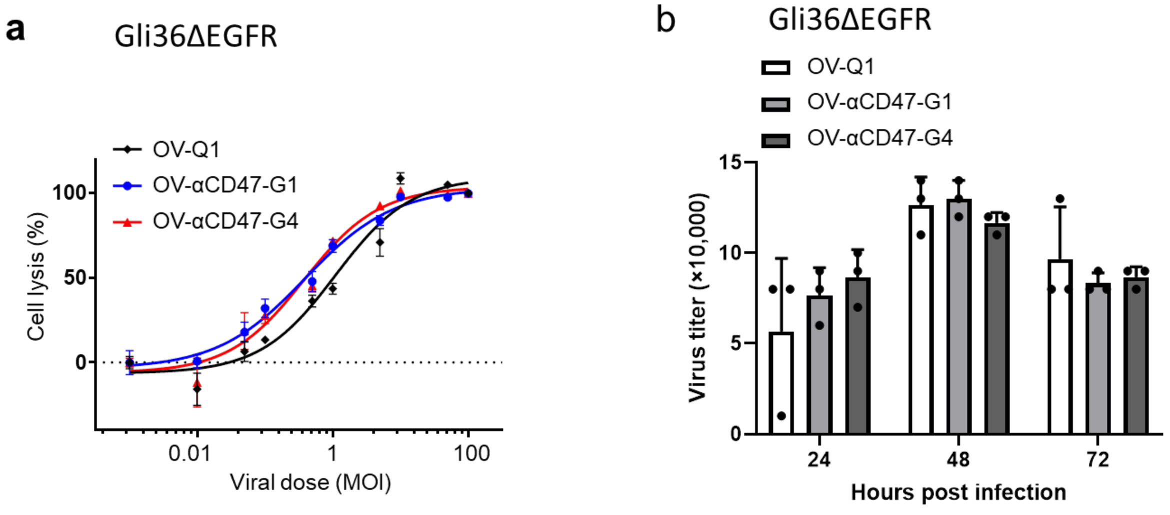

Supplementary Figure 3. Viral production and infectivity of OV-αCD47-G1 and OV-αCD47-G4. (**a**) Gli36ΔEGFR human GBM cells were infected with OV-Q1, OV-αCD47-G1, or OV-αCD47-G4 at the indicated MOIs. Cell lysis was analyzed 3 days after infection by CCK8 assay. Data are presented as mean values +/- SD (n = 3). (**b**) Gli36ΔEGFR human GBM cells were infected with OV-Q1, OV-αCD47-G1, or OV-αCD47-G4 at an MOI of 2. The supernatants were harvested at indicated time points and checked for viral reproduction using a plaque assay in Vero cells. Data are representative of three independent experiments with similar data. Data are presented as mean values +/- SD (n = 3). No statistical differences were noted.

Supplementary Figure 4

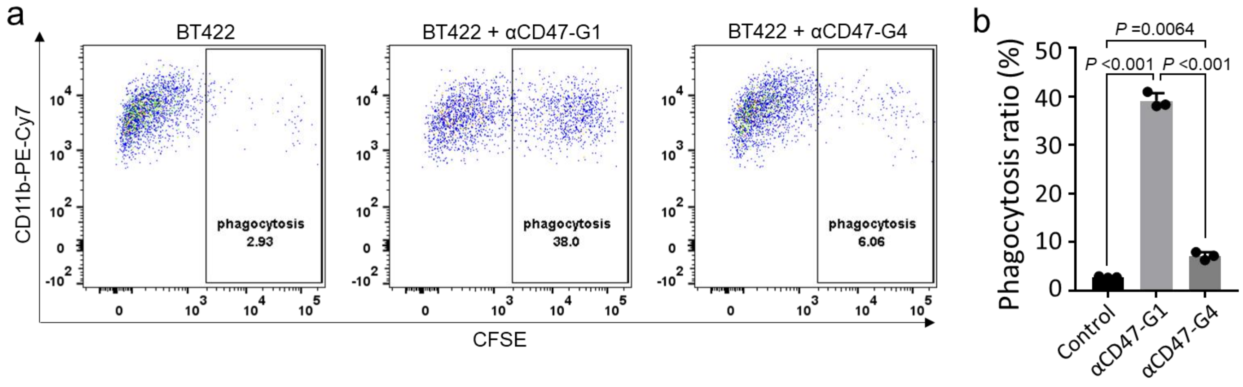

Supplementary Figure 4. αCD47-G1 and αCD47-G4 induce phagocytosis of GBM cells by mouse BMDMs. The effect of αCD47-G1 and αCD47-G4 purified from CHO cells on phagocytosis of GBM cells by mouse bone marrow derived-macrophages (BMDMs). (**a & b**) BT422 human GBM cells were labeled with CFSE and co-cultured with mouse BMDMs at an effector:target ratio of 1:2 in the presence of vehicle control, αCD47-G1 or αCD47-G4 at a concentration of 5 μg/ml. Phagocytosis was quantified by the percentage of BMDM uptake of labeled tumor cells (CD11b+CFSE+). Data presented in (a) are representative results and in (b) are summarized results. One-way ANOVA with P values corrected for multiple comparisons by the Bonferroni test (n = 3 technical replicates). All experiments were performed with three mice per group. Data are presented as mean values +/- SD (n = 3).

Supplementary Figure 5

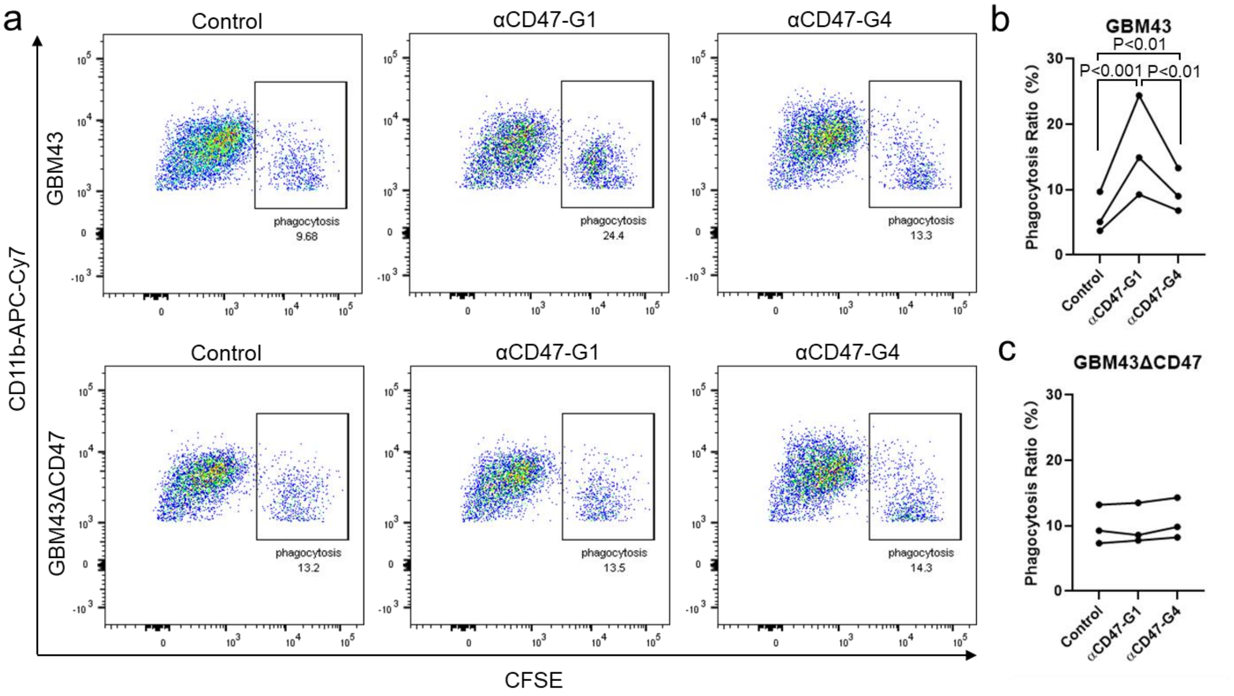

Supplementary Figure 5. Macrophage phagocytosis with GBM43ΔCD47 and GBM43 as target cells. (a-c) The effect of αCD47-G1 and αCD47-G4 purified from CHO cells on phagocytosis of GBM43 and GBM43ΔCD47 cells by human macrophages. GBM43 and GBM43ΔCD47 cells were labeled with CFSE and co-cultured with human macrophages at an effctor:target ratio of 1:2 in the presence of vehicle control, αCD47-G1 or αCD47-G4 at a concentration of 5 μg/ml. Phagocytosis was quantified by the percentage of human macrophage uptake of labeled tumor cells (CD11b+CFSE+). Data presented in (a) are representative results and in (b and c) are summarized results. One-way ANOVA with P values corrected for multiple comparisons by the Bonferroni method after data log transformation (n = 3 donors).

Supplementary Figure 6

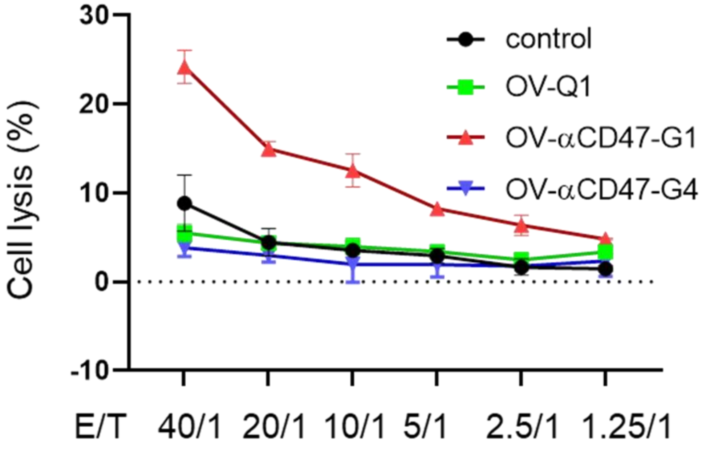

Supplementary Figure 6. αCD47-G1 but not αCD47-G4 induces cytotoxicity of human NK cells against GBM cells at various effector: target ratios. Cytotoxicity of human primary NK cells against GBM43 cells in control media or conditioned media from the culture of OV-Q1-, OV-αCD47-G1- or OV-αCD47-G4-infected U251T2 GBM cells at various effector (E): target (T) ratios between 40:1 and 1.25:1 in a ^{51}Cr release assay. $P < 0.001$, OV-αCD47-G1 vs. control at an E:T ratio of 2.5. One-way ANOVA with P values corrected for multiple comparisons by the Bonferroni test. Error bars represent standard deviations of means of three donors.

Supplemental Figure 7

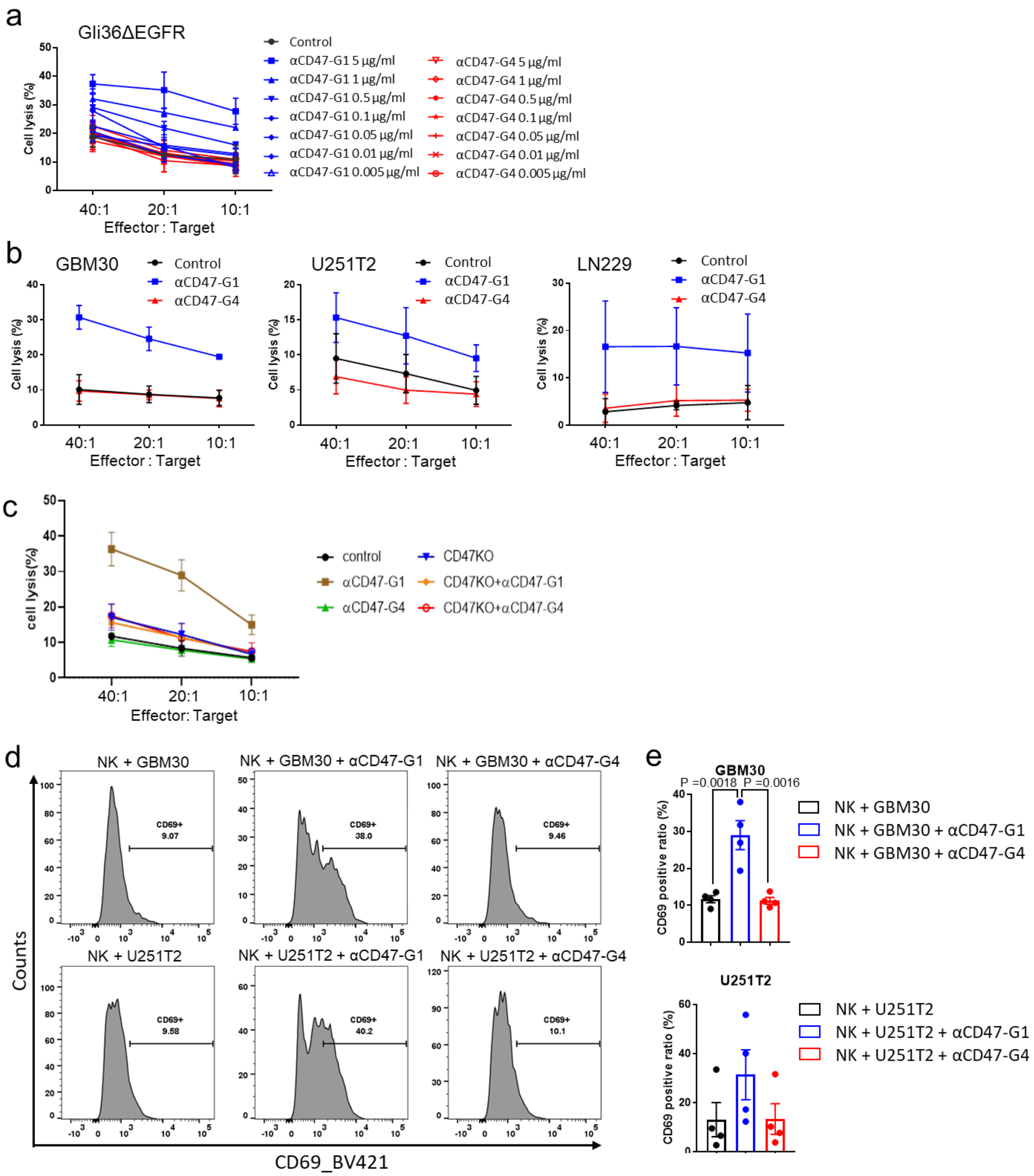

Supplementary Figure 7. αCD47-G1 but not αCD47-G4 induces cytotoxicity of human NK cells against GBM cells. (a) Cytotoxicity of human primary NK cells against Gli36ΔEGFR human GBM cells pretreated with different doses of αCD47-G1 or αCD47-G4 for 20 min. NK cells were added to the pre-treated GBM cells without washing at the effector: target ratios of 40:1, 20:1, and 10:1 in a ^{51}Cr release assay. **(b)** Cytotoxicity of primary human NK cells against

αCD47-G1- and αCD47-G4-pretreated three human GBM lines: GBM30, U251T2, and LN229. A linear mixed model was used to account for the underlying variance and covariance structure (n = 4 donors). **(c)** NK cell killing assay with GBM43ΔCD47 and GBM43 as target cells. For GBM43 cells as target cells, αCD47-G1 vs. control, $P < 0.001$. αCD47-G4 vs. control, no significance. For GBM43 ΔCD47 as target cells, αCD47-G1 vs. control, no significance. αCD47-G4 vs. control, no significance. A linear mixed model was used to account for the underlying variance and covariance structure. The experiments were performed with three donors in triplicate. Error bars represent standard deviations of means of three donors. A linear mixed model was used to account for the underlying variance and covariance structure (n = 4 donors) **(d)** αCD47-G1 but not αCD47-G4 induced the expression of the NK cell activation marker CD69. GBM30 or U251T2 human GBM cells that were pretreated with αCD47-G1 or αCD47-G4 at the concentration of 1 µg/ml and then co-cultured with primary human NK cells at a ratio of 1:1 for 4 hours. No cell wash was applied before NK cells were added. CD69 expression on NK cells was measured by flow cytometry to quantify the level of NK cell activation. **(e)** Statistic summary of (d). One-way ANOVA with Bonferroni's multiple comparisons test (n = 4 donors). The experiments were performed with four donors in triplicate. Error bars represent standard deviations of means of four donors.

Supplementary Figure 8

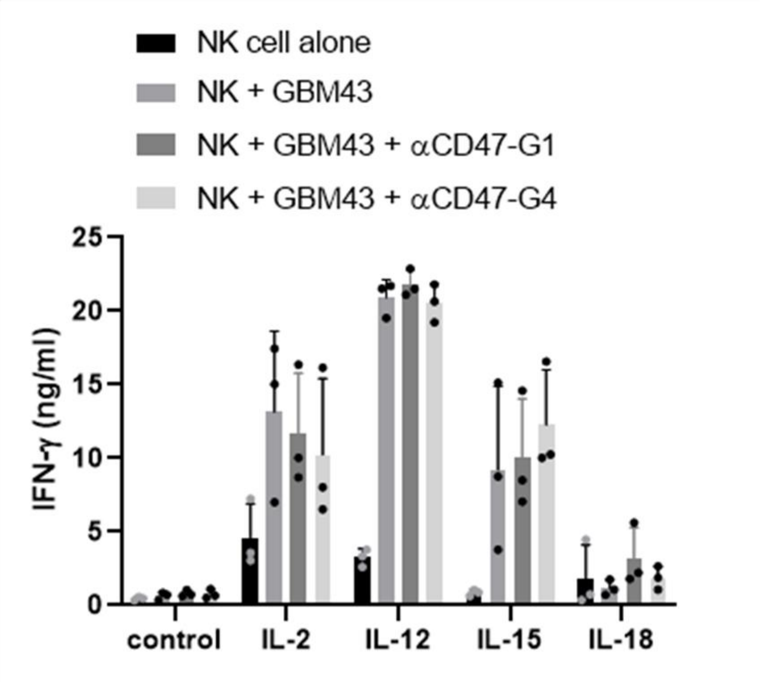

Supplementary Figure 8. IFN-γ ELISA assay. Primary human NK cells were co-cultured with GBM43 cells with αCD47-G1 or αCD47-G4 in the presence of IL-2, IL-12, IL-15, IL-18, or PBS vehicle control. The experiments were performed with three donors in triplicate. Error bars represent standard deviations of means of three donors.

Supplementary Figure 9

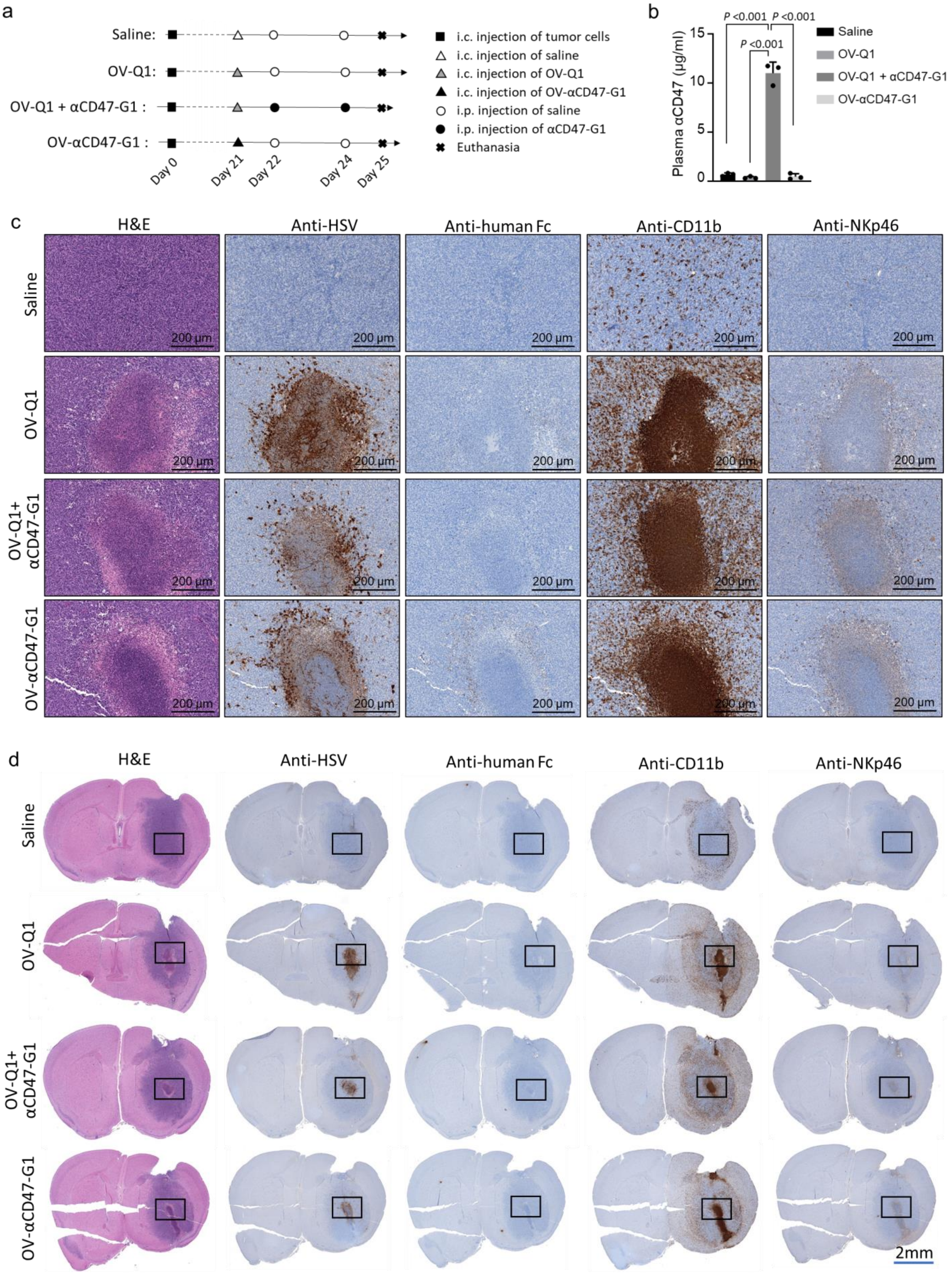

Supplementary Figure 9. OV-αCD47-G1 infection continuously delivers anti-CD47 antibody to the tumor microenvironment, while systemic infusion of αCD47-G1 does not. (**a**) Experimental timeline for *in vivo* studies using an orthotopic model of human GBM. Details of the experiment are provided in the Method section. There were four treatment groups: Group 1, saline; Group 2, OV-Q1; Group 3, a combination of OV-Q1 plus i.p. administration of αCD47-G1; and Group 4, OV-αCD47-G1. On day 0, mice were implanted with 1×10^5 GBM43 cells i.c.. After tumor implantation, Groups 2, 3, and 4 received intratumoral injection of oHSV (OV-Q1 or OV-αCD47-G1) at a dose of 2×10^5 PFU per mouse on day 21. Group 1 received saline as control. On days 22 and 24, Group 3 received i.p. injection of purified αCD47-G1 at the dose of 150 µg per mouse per day. Groups 1, 2, and 4 received i.p. injections of saline as control. All mice were euthanized on day 25 for blood and brain harvesting. **(b)** The concentration of αCD47-G1 in plasma was measured by ELISA in mice from different treatments. One-way ANOVA with P values corrected for multiple comparisons by Bonferroni method multiple comparisons test (n = 3 mice). **(c & d)** Slides from the brains isolated from experimental mice were subjected to H&E staining and immunohistochemical staining (IHC). IHC was performed with anti-HSV, anti-human Fc, which identifies αCD47-G1, anti-CD11b, or anti-NKp46 antibodies. High and low magnifications of brain H&E and IHC with anti-HSV, anti-human Fc, anti-CD11b, and anti-Nkp46 staining at the tumor implantation site are shown in (c) and (d), respectively. The images with high magnification in (c) are exactly are representative of the boxed area in (d). Data presented are represented of one (c & d) or three (b) mice of at least three mice in total with similar data. Error bars (b) represent standard deviations of means of three mice.

Supplementary Figure 10

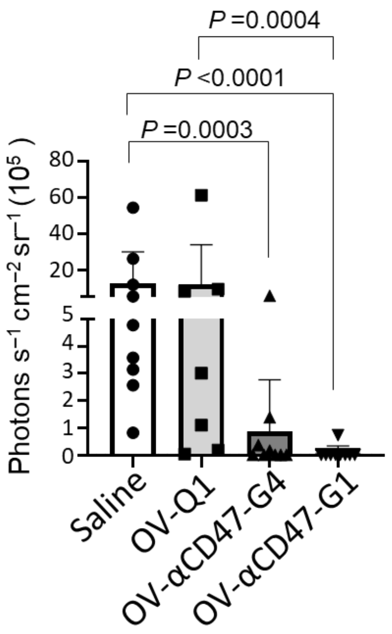

Supplementary Figure 10. Quantification of the luciferase expression in the GBM43-FFL mouse model. The luciferase intensity of the experimental mice shown in the main Figure 5b was measured 35 days post tumor implantation. Data are presented as mean values +/- SD. One-way ANOVA with P values corrected for multiple comparisons by the Bonferroni test. (n = 7 for OV-Q1 and 9 for other groups).

Supplementary Figure 11

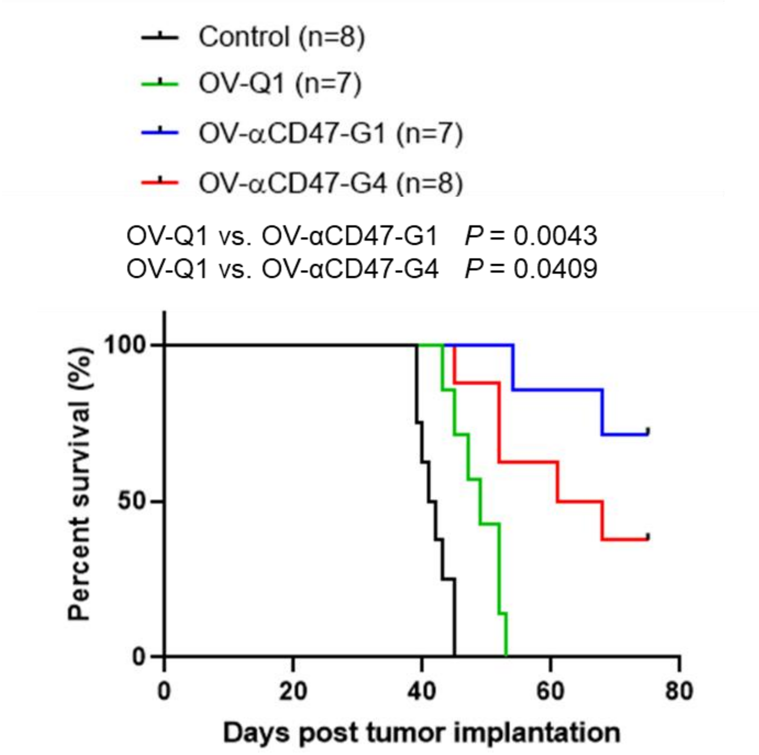

Supplementary Figure 11. The therapeutic effects of OV-αCD47-G1 and OV-αCD47-G4 on treating mice with xenograft GBM. The xenograft GBM model was established by intracranial (i.c.) injection of 1×10^5 GBM43 cells. Seven days after tumor implantation, mice were received an i.c. injection with OV-αCD47-G1, OV-αCD47-G4 or OV-Q1 at the dose of 2×10^5 PFU per mouse, or saline as a placebo control. Survival was estimated by the Kaplan–Meier method and compared by two-side log-rank test (n = 7 or 8 animals).

Supplementary Figure 12

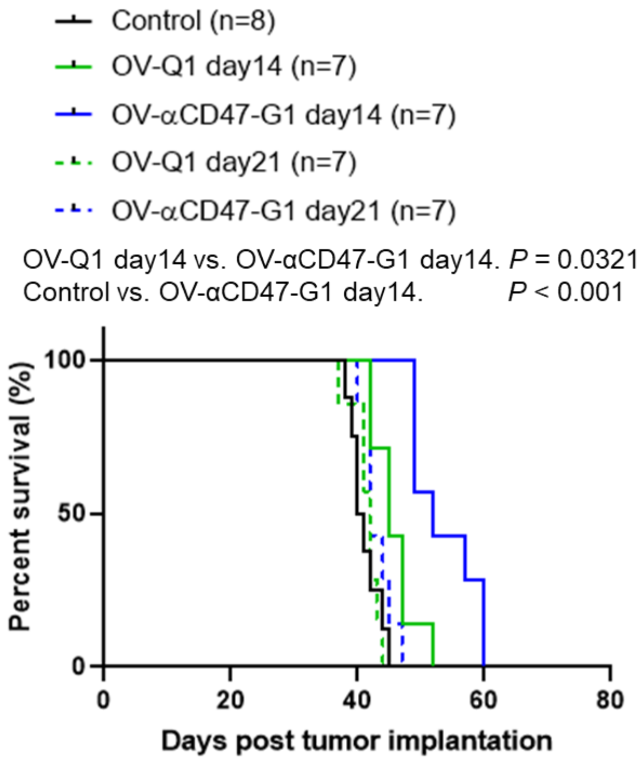

Supplementary Figure 12. The therapeutic effect of OV-αCD47-G1 on treating an established GBM model with big tumors. The xenograft GBM model was established by i.c. injection of 1×10^5 GBM43 cells. Fourteen or twenty-one days after tumor implantation, mice were received an i.c. injection with OV-αCD47-G1 or OV-Q1 at the dose of 2×10^5 PFU per mouse, or saline as a placebo control. Survival was estimated by the Kaplan–Meier method and compared by two-side log-rank test (n = 7 or 8 animals per group).

Supplementary Figure 13

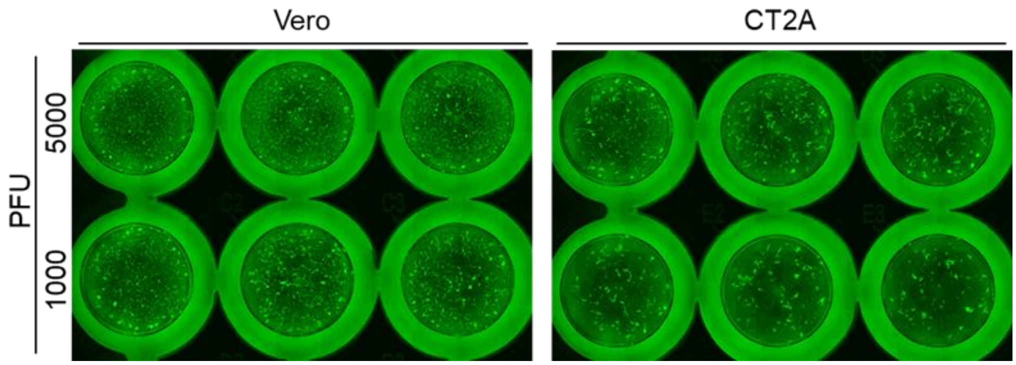

Supplementary Figure 13. Susceptibility of CT2A cells to OV-Q1 infection. Vero cells were used as positive control for comparison of susceptibility of CT2A to OV-Q1.

Supplementary Figure 14

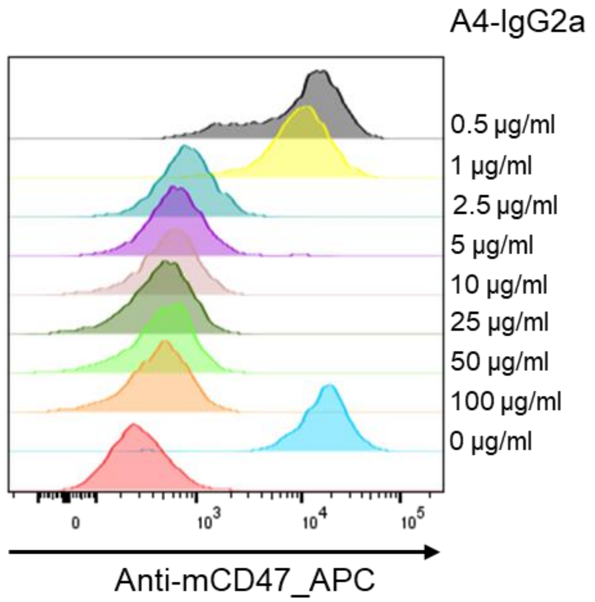

Supplementary Figure 14. The blocking effect of A4-IgG2b. CT2A cells were incubated with an increasing concentration of unlabeled A4-IgG2b mAb, followed by incubation with an APC-conjugated anti-mouse CD47 antibody. The blocking effect was assessed by flow cytometry.

Supplementary Figure 15

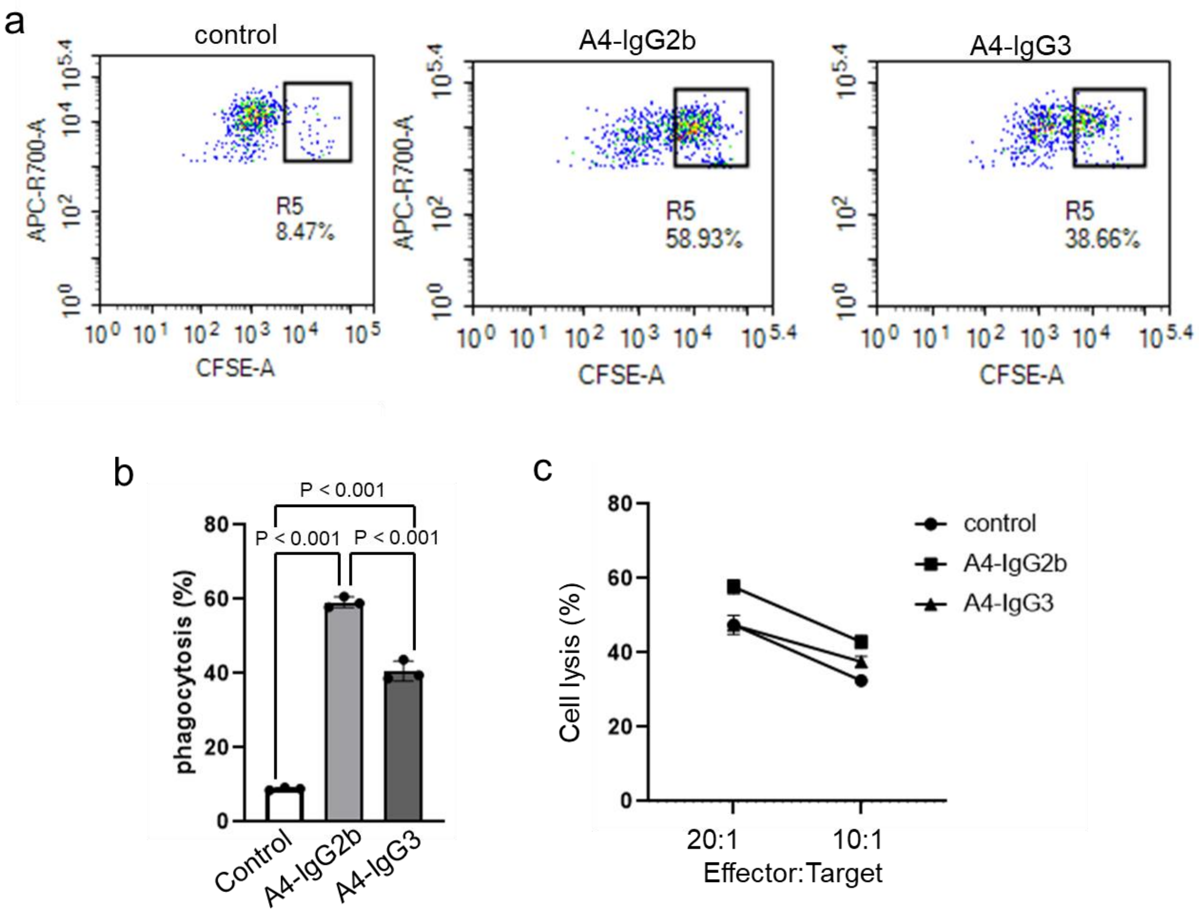

Supplementary Figure 15. The effect of A4-IgG2b and A4-IgG3 on macrophage and NK cell activation. (a & b) CT2A cells were labeled with CFSE and co-cultured with mouse BMDMs at an effector:target ratio of 1:2 in the presence of PBS vehicle control, A4-IgG2b, or A4-IgG3 at a concentration of 5 μg/ml. Phagocytosis was quantified by the percentage of BMDM uptake of labeled tumor cells (CD11b+CFSE+). Data in (a) are representative results and in (b) are summarized results. One-way ANOVA with P values corrected for multiple comparisons by the Bonferroni test. Data are presented as mean values +/- SD (n = 3). All experiments were performed with three mice per group. Error bars represent standard deviations of three mice. **(c)** Cytotoxicity of mouse NK cells against CT2A cells pre-treated with A4-IgG2b or A4-IgG3 for 20 min. NK cells were added to the pre-treated GBM cells without washing at the effector: target ratios of 20:1 and 10:1 in a ^{51}Cr release assay. A4-IgG2b vs. control, $P < 0.01$; A4-IgG3 vs. control, no significance. A linear mixed model was used to account for the underlying variance and covariance structure.

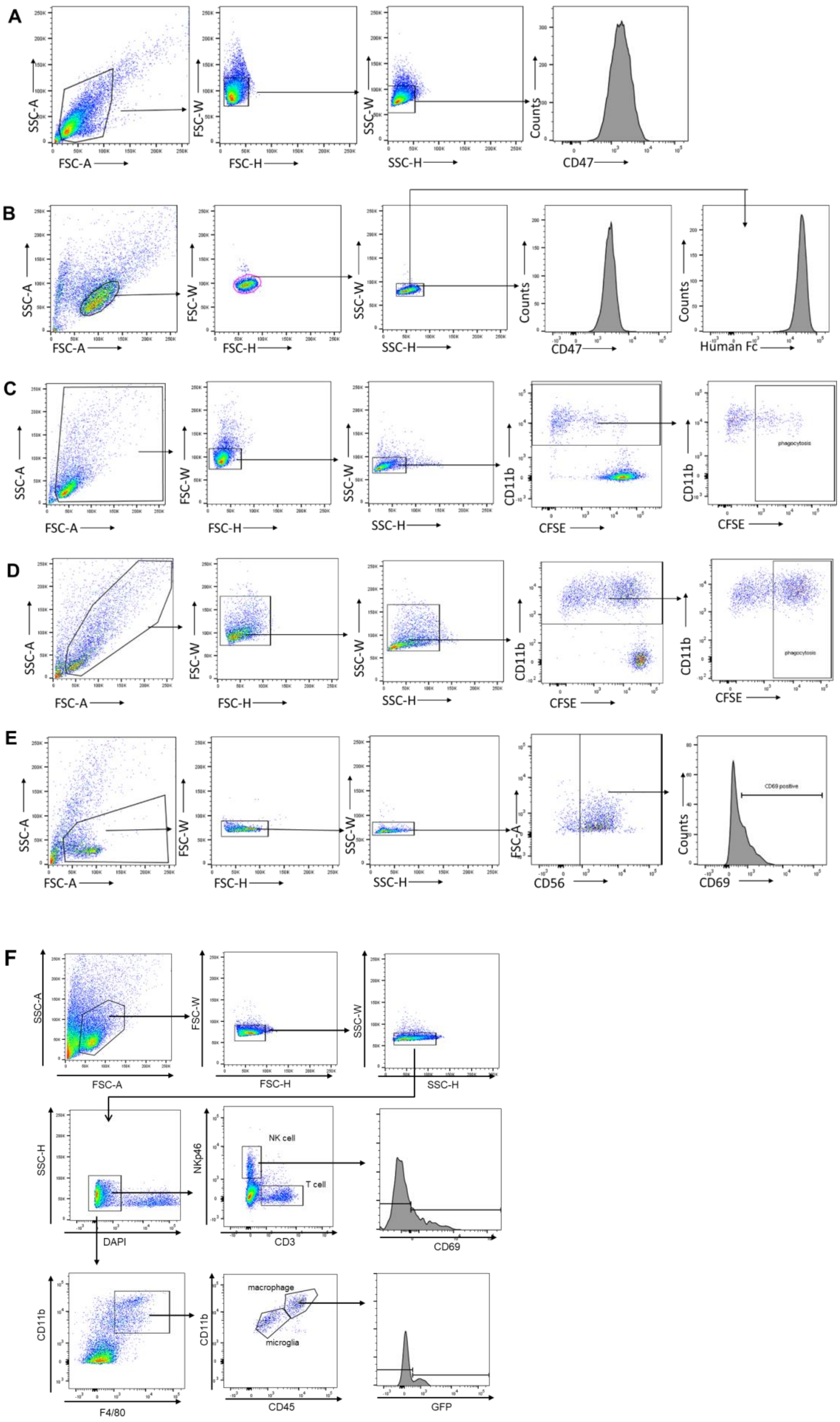
A
SSC-A
FSC-A
FSC-W
FSC-H
SSC-W
SSC-H
Counts
CD47
B
Human Fc
C
CD11b
CFSE
phagocytosis
D
E
CD56
CD69
CD69 positive
F
DAPI
NKp46
CD3
NK cell
T cell
F4/80
CD45
macrophage
microglia
GFP

Supplementary Figure 16. Flow cytometry gating strategy. (a) To determine CD47 expression in GBM cells, live cells were further gated on FSC-A/SSC-A, FSC-H/FSC-W, and then SSC-H/SSC-W events to check surface expression levels of CD47 (for Fig. 1a and Supplementary Fig. 1). **(b)** For CD47 binding and blocking assays, live cells were further gated on FSC-A/SSC-A, FSC-H/FSC-W, and then SSC-H/SSC-W events to check surface expression levels of CD47 and human Fc (for Fig. 1b and c). **(c & d)** For the phagocytosis assay of macrophages, live cells were further gated on FSC-A/SSC-A, FSC-H/FSC-W, and then SSC-H/SSC-W events. Macrophages were defined as CD11b(+). Phagocytosis was measured as the percentage of CD11b(+) CFSE(+) macrophages (for Fig. 2a, c, g and i). **(e)** To determine CD69 expression in primary human NK cells, live cells were further gated on FSC-A/SSC-A, FSC-H/FSC-W, and then SSC-H/SSC-W events. NK cells were defined as CD56(+) events to check surface expression levels of CD69 (for Fig. 3d and Supplementary Fig. 5c). **(f)** For flow cytometric assays on murine immune cells, live cells were gated on FSC-A/SSC-A, FSC-H/FSC-W, SSC-H/SSC-W, and then SSC-H/DAPI. NK cells were defined as NKp46(+)CD3(-) (for Fig. 7d, g and h); Macrophages were defined as $CD45^{high}$CD11b(+)F4/80(+) (for Fig. 7e, i and j); T cells were defined as NKp46(-)CD3(+) (for Fig. 7f).

Supplementary Tables

Supplementary Table 1. Antibodies used in this study

Item	Catalogue	Vendor	Dilution ratio
BV786 Mouse Anti-Human CD47	563758	BD biosciences	5 µl/test
BV421 Mouse Anti-Human CD69	562883	BD biosciences	5 µl/test
PE-Cy™7 Rat Anti-CD11b	552850	BD biosciences	5 µl/test
Alexa Fluor® 700 Mouse Anti-Human CD56	557919	BD biosciences	5 µl/test
Alexa Fluor® 647 AffiniPure Mouse Anti-Human IgG, Fcγ fragment specific	209-605-098	Jackson ImmunoResearch	5 µl/test
PE/Cyanine7 anti-mouse CD335 (NKp46) Antibody	137618	Biolegend	5 µl/test
APC Hamster Anti-Mouse CD3e (RUO)	553066	BD biosciences	5 µl/test
APC Rat Anti-Mouse CD45 (RUO)	559864	BD biosciences	5 µl/test
PE-Cy™7 Rat Anti-CD11b	552850	BD biosciences	5 µl/test
F4/80 Monoclonal Antibody (BM8), PE, eBioscience	12-4801-82	Thermo Fisher	5 µl/test
BV786 Hamster Anti-Mouse CD69 (RUO)	564683	BD biosciences	5 µl/test
BV510 Mouse Anti-Human Granzyme B (RUO)	563388	BD biosciences	5 µl/test
Anti-human IgG heavy chain	MAB1307	sigma	1:500
Anti-HSV	361A-15-ASR	Cell marque	1:100
Anti-human Fc	109-005-098	Jackson ImmunoResearch	1:100
Anti-CD11b	ab133357	abcam	1:100
Anti-NKp46	AF2225	R&D System	1:100

Supplementary Table 2. Primers used in this study

Name	Sequence
mIl6 F	CTGCAAGAGACTTCCATCCAG
mIl6 R	AGTGGTATAGACAGGTCTGTTGG
mArg1 F	CTCCAAGCCAAAGTCCTTAGAG
mArg1 R	GGAGCTGTCATTAGGGACATCA
mCcl2 F	ATCCACGGCATACTATCAACATC
mCcl2 R	TCGTAGTCATACGGTGTGGTG
mCcl4 F	CCAGCTCTGTGCAAACCTAACC
mCcl4 R	GCCACGAGCAAGAGGAGAGA
mNos2 f	TGACGGCAAACATGACTTCAG
mNos2 R	GGTGCCATCGGGCATCT
mIl10 F	CAGTACAGCCGGGAA GACAATAA
mIl10 R	CCGCAGCTCTAGGAGCATGT
mIl12b F	CCTGAAGTGTGAAGCACCAAATT
mIl12b R	CTTCAAGTCCATGTTTCTTTGCA
mIl1b F	GAAATGCCACCTTTTGACAGTG
mIl1b R	TGGATGCTCTCATCAGGACAG
hIL1B F	TTCGACACATGGGATAACGAGG
hIL1B R	TTTTTGCTGTGAGTCCCGGAG
hIL6 F	CCTGAACCTTCCAAAGATGGC
hIL6 R	TTCACCAGGCAAGTCTCCTCA
hIL12A F	ATGGCCCTGTGCCTTAGTAGT
hIL12A R	AGCTTTGCATTCATGGTCTTGA
hNOS2 F	AGGGACAAGCCTACCCCTC
hNOS2 R	CTCATCTCCCGTCAGTTGGT
hIL10 F	GACTTTAAGGGTTACCTGGGTTG
hIL10 R	TCACATGCGCCTTGATGTCTG
18s rRNA F	GTAACCCGTTGAACCCCATT
18s rRNA R	CCATCCAATCGGTAGTAGCG
