# Supplementary material for: An oncolytic virus expressing a full-length antibody enhances antitumor innate immune response to glioblastoma
Source: Nat Commun. 2021 Oct 8;12:5908. doi: 10.1038/s41467-021-26003-6 (PMC8501058; doi:10.1038/s41467-021-26003-6)
Supplement: Supplementary file 2 — Reporting Summary [file 41467_2021_26003_MOESM2_ESM.pdf]

nature portfolio

Corresponding author(s): Prof. Michael A Caligiuri

Last updated by author(s): Aug 12, 2021

Reporting Summary

Nature Portfolio wishes to improve the reproducibility of the work that we publish. This form provides structure for consistency and transparency in reporting. For further information on Nature Portfolio policies, see our Editorial Policies and the Editorial Policy Checklist.

Statistics

For all statistical analyses, confirm that the following items are present in the figure legend, table legend, main text, or Methods section.

n/a	Confirmed	
☐	☒	The exact sample size (*n*) for each experimental group/condition, given as a discrete number and unit of measurement
☐	☒	A statement on whether measurements were taken from distinct samples or whether the same sample was measured repeatedly
☐	☒	The statistical test(s) used AND whether they are one- or two-sided<br>*Only common tests should be described solely by name; describe more complex techniques in the Methods section.*
☒	☐	A description of all covariates tested
☐	☒	A description of any assumptions or corrections, such as tests of normality and adjustment for multiple comparisons
☐	☒	A full description of the statistical parameters including central tendency (e.g. means) or other basic estimates (e.g. regression coefficient) AND variation (e.g. standard deviation) or associated estimates of uncertainty (e.g. confidence intervals)
☐	☒	For null hypothesis testing, the test statistic (e.g. *F*, *t*, *r*) with confidence intervals, effect sizes, degrees of freedom and *P* value noted<br>*Give P values as exact values whenever suitable.*
☒	☐	For Bayesian analysis, information on the choice of priors and Markov chain Monte Carlo settings
☒	☐	For hierarchical and complex designs, identification of the appropriate level for tests and full reporting of outcomes
☒	☐	Estimates of effect sizes (e.g. Cohen's *d*, Pearson's *r*), indicating how they were calculated

Our web collection on statistics for biologists contains articles on many of the points above.

Software and code

Policy information about availability of computer code

Data collection	No customized software was used; BD FACSDiva version 6 was used to collect flow cytometric data; microscope data were collected using Zeiss ZEN. Western blot data were collected by Odyssey CLx system. Additional information about software was described in the manuscript or available upon request.
Data analysis	No custom-made software was used for data analysis. Statistical software GraphPad, R.3.6.3. and SAS 9.4 were used for the statistical analysis. The Microscope images were analyzed using Zeiss ZEN (blue edition). Flow cytometry data were analyzed using Flowjo 7.6 or 10.0.

For manuscripts utilizing custom algorithms or software that are central to the research but not yet described in published literature, software must be made available to editors and reviewers. We strongly encourage code deposition in a community repository (e.g. GitHub). See the Nature Portfolio guidelines for submitting code & software for further information.

Data

Policy information about availability of data

All manuscripts must include a data availability statement. This statement should provide the following information, where applicable:

- Accession codes, unique identifiers, or web links for publicly available datasets
- A description of any restrictions on data availability
- For clinical datasets or third party data, please ensure that the statement adheres to our policy

All original data underlying selected data shown in the figures and supplemental figures are available from the corresponding author upon reasonable request. Source data are provided with this paper.

Field-specific reporting

Please select the one below that is the best fit for your research. If you are not sure, read the appropriate sections before making your selection.

☒ Life sciences ☐ Behavioural & social sciences ☐ Ecological, evolutionary & environmental sciences

For a reference copy of the document with all sections, see nature.com/documents/nr-reporting-summary-flat.pdf

Life sciences study design

All studies must disclose on these points even when the disclosure is negative.

Sample size	No statistical method was used to predetermine the sample size. Sample sizes in this study were estimated based on previous experience that showed significance (PMID: 30475349). For in vitro studies, the experiments were repeated at least 3 times with samples size at least 3 to control technical variations. For the experiments that using primary human NK cells or macrophages, experiments were repeated with at least 3 different donors to control biological variations. For survival studies, at least 6 animals for each group were used to obtain informative results.
Data exclusions	No data were excluded for all figures.
Replication	All experiments were reliably reproduced and results are represented as mean +/-SD as appropriate. One-way ANOVA model was utilized to compare three or more conditions. For data with repeated measures from the same donor, linear mixed model was utilized to account for the variance-covariance structure due to repeated measures. When needed, P values were adjusted for multiple comparisons using Holm's or the Bonferroni method procedure. A P value of 0.05 or less was considered statistically significant, which is described in the methods section of the main text.For in vitro studies, the experiments were repeated at least 3 times with samples size at least 3 to control technical variations.
Randomization	Peripheral blood cones used to isolate NK cells or macrophages were de-identified and randomly picked up. After the establishment of GBM mouse model, animals were randomly grouped for in vivo experiments. Throughout the whole experiment, samples and animals were randomized into groups.
Blinding	For the survival study, the investigators were blinded to the treatment. No blinding was used in vitro experiments. All data collected was quantifiable and blinding would not change any bias in data collected.

Reporting for specific materials, systems and methods

We require information from authors about some types of materials, experimental systems and methods used in many studies. Here, indicate whether each material, system or method listed is relevant to your study. If you are not sure if a list item applies to your research, read the appropriate section before selecting a response.

Materials & experimental systems

n/a	Involved in the study	
☐	☒	Antibodies
☐	☒	Eukaryotic cell lines
☒	☐	Palaeontology and archaeology
☐	☒	Animals and other organisms
☐	☒	Human research participants
☒	☐	Clinical data
☒	☐	Dual use research of concern

Methods

n/a	Involved in the study	
☒	☐	ChIP-seq
☐	☒	Flow cytometry
☒	☐	MRI-based neuroimaging

Antibodies

Antibodies used	Antibodies used for flow cytometry:<br>BV786 Mouse Anti-Human CD47 (BD Biosciences, Cat#563758, clone: B6H12, lot: 7283921, 5 µl per test)<br>BV421 Mouse Anti-Human CD69 (BD Biosciences, Cat#562883, clone:FN50, lot:9038961, 5 µl per test)<br>PE-Cy™7 Rat Anti-CD11b (BD Biosciences, Cat#552850, clone:M1/70, lot:B279980, 5 µl per test)<br>Alexa Fluor® 700 Mouse Anti-Human CD56 (BD Biosciences, Cat#557919, lot:9038961, 5 µl per test)<br>Alexa Fluor® 647 AffiniPure Mouse Anti-Human IgG, Fcγ fragment specific (Jackson ImmunoResearch, Cat#209-605-098, lot:137557, 5 µl per test)<br>APC Rat Anti-Mouse CD45 (RUO) (BD biosciences, Cat#559864,, 5 µl per test),<br>PE-Cy™7 Rat Anti-CD11b (BD biosciences, Cat#552850, 5 µl per test),<br>F4/80 Monoclonal Antibody (Thermo Fisher, Cat#12-4801-82, 5 µl per test)<br>BV786 Hamster Anti-Mouse CD69 (BD biosciences, Cat#564683, 5 µl per test)<br>BV510 Mouse Anti-Human Granzyme B (BD biosciences, Cat#563388, 5 µl per test) .<br>Antibody used for blocking phagocytosis assay:

Ultra-LEAF™ Purified Human IgG1 Isotype Control Recombinant Antibody(Biolegend, Cat#403505, clone: QA16A12, lot: B293925, 5 μl per test)
Antibodies used for immunoprecipitation:
Anti-human IgG heavy chain (sigma, Cat#MAB1307,clone: HP6001, dilution ratio:1:500)
Anti-human kappa chain (Thermo fisher, MA5-12117, clone: L1C1, dilution ratio:1:500)
Antibodies used for Immunohistochemistry assay:
Anti-HSV (Cell marque, Cat#361A-15-ASR, dilution ratio:1:100),
Anti-human Fc (Jackson ImmunoResearch, Cat#109-005-098 ,dilution ratio:1:100)
Anti-CD11b (abcam, Cat#ab133357, dilution ratio:1:100)

Validation

Antibodies used for flow cytometry:
BV786 Mouse Anti-Human CD47 (BD, Cat#563758, clone: B6H12, lot: 7283921).Please see the manufacturer's website link for application. https://www.bdbiosciences.com/us/applications/research/stem-cell-research/cancer-research/human/bv786-mouseanti-human-cd47-b6h12/p/563758
BV421 Mouse Anti-Human CD69 (BD, Cat#562883, clone:FN50, lot:9038961).Please see the manufacturer's website link for application. https://www.bdbiosciences.com/us/applications/research/b-cell-research/surface-markers/human/bv421-mouse-antihuman-cd69-fn50-also-known-as-fn-50/p/562883
PE-Cy™7 Rat Anti-CD11b (BD, Cat#552850, clone:M1/70, lot:B279980).Please see the manufacturer's website link for application. https://www.bdbiosciences.com/us/applications/research/stem-cell-research/mesenchymal-stem-cell-markers-bone-marrow/mouse/negative-markers/pe-cy7-rat-anti-cd11b-m170/p/552850
Alexa Fluor® 700 Mouse Anti-Human CD56 (BD, Cat#557919, lot:9038961).Please see the manufacturer's website link for application. https://www.bdbiosciences.com/us/applications/research/stem-cell-research/hematopoietic-stem-cell-markers/human/negativemarkers/alexa-fluor-700-mouse-anti-human-cd56-b159/p/557919
Alexa Fluor® 647 AffiniPure Mouse Anti-Human IgG, Fcγ fragment specific (Jackson ImmunoResearch, Cat#209-605-098, lot:137557) Please see the manufacturer's website link for application. https://www.jacksonimmuno.com/catalog/products/109-605-098
Antibody used for blocking phagocytosis assay:
Ultra-LEAF™ Purified Human IgG1 Isotype Control Recombinant Antibody(Biolegend, Cat#403505, clone: QA16A12, lot: B293925).Please see the manufacturer's website link for application. https://www.biolegend.com/en-us/products/apc-human-igg1-isotype-control-recombinant-antibody-15053
PE/Cyanine7 anti-mouse CD335 (NKp46) Antibody (Biolegend, Cat#137618, clone: 29A1.4, lot:b278827). Please see the manufacturer's website link for application. https://www.biolegend.com/en-us/products/pe-cyanine7-anti-mouse-cd335-nkp46-antibody-7899?GroupID=BLG8849
APC Hamster Anti-Mouse CD3e (RUO) (BD, Cat#553066, clone:145-2C11, lot:9065910). Please see the manufacturer's website link for application. https://www.bdbiosciences.com/us/applications/research/t-cell-immunology/th-1-cells/surface-markers/mouse/apchamster-anti-mouse-cd3e-145-2c11/p/553066.
APC Rat Anti-Mouse CD45 (RUO) (BD, Cat#559864, Clone: 30-F11, lot:8277680).Please see the manufacturer's website link for application. https://www.bdbiosciences.com/eu/applications/research/stem-cell-research/cancer-research/mouse/apc-rat-antimouse-cd45-30-f11/p/559864
PE-Cy™7 Rat Anti-CD11b (RUO)(BD, Cat#552850, Clone: M1/70, lot:9311513). Please see the manufacturer's website link for application. https://www.bdbiosciences.com/eu/applications/research/stem-cell-research/mesenchymal-stem-cell-markers-bonemarrow/mouse/negative-markers/pe-cy7-rat-anti-cd11b-m170/p/552850
F4/80 Monoclonal Antibody (BM8), PE, eBioscience (Thermo Fisher, Cat#12-4801-82, clone:BM8, lot:2049425). Please see the manufacturer's website link for application. https://www.thermofisher.com/cn/zh/antibody/product/F4-80-Antibody-clone-BM8-Monoclonal/12-4801-82
BV786 Hamster Anti-Mouse CD69 (RUO)(BD, Cat#564683, Clone:H1.2F3, lot: 8282705). Please see the manufacturer's website link for application. https://www.bdbiosciences.com/us/applications/research/b-cell-research/surface-markers/mouse/bv786-hamsteranti-mouse-cd69-h12f3/p/564683
BV510 Mouse Anti-Human Granzyme B (RUO)(BD, Cat#563388, clone:GB11, lot:9093962). Please see the manufacturer's website link for application. https://www.bdbiosciences.com/us/applications/research/t-cell-immunology/regulatory-t-cells/intracellularmarkers/cytokines-and-chemokines/human/bv510-mouse-anti-human-granzyme-b-gb11/p/563388
Antibodies used for immunoprecipitation:
Anti-human IgG heavy chain (sigma, Cat#MAB1307,clone: HP6001).Please see the manufacturer's website link for application. https://www.emdmillipore.com/US/en/product/Mouse-Anti-Human-IgG1-Antibody-clone-HP6001-Fc,MM_NF-MAB1307
Anti-human kappa chain (Thermo fisher, MA5-12117, clone: L1C1).Please see the manufacturer's website link for application. MA5-12117
Antibodies used for Immunohistochemistry assay:
Anti-HSV (Cell marque, 361A-15-ASR), Please see the manufacturer's website link for application. http://www.cellmarque.com/antibodies/CM/1948/Herpes-Simplex-Virus-I_polyclonal
Anti-human Fc (Jackson ImmunoResearch, 109-005-098), Please see the manufacturer's website link for application. https://www.jacksonimmuno.com/catalog/products/109-005-098
Anti-CD11b (abcam, ab133357). Please see the manufacturer's website link for application. https://www.abcam.com/cd11bantibody-epr1344-ab133357.html
Anti-NKp46 (R&D System, AF2225). Please see the manufacturer's website link for application. https://www.rndsystems.com/cn/products/mouse-nkp46-ncr1-antibody_af2225

Eukaryotic cell lines

Policy information about cell lines

Cell line source(s)

Gli36, U251, LN229, GBM30, Vero and CT2A cells were obtained from the laboratory of E. Antonio Chiocca.
GBM43 and BT422 were developed at Mayo Clinic. The original commercial source of Vero cells, U251, and LN229 is ATCC.
CT2A cells were originally purchased from MilliporeSigma.
Primary human NK cells and macrophages were isolated from cones of health donors from City of Hope National Medical Center.

Authentication	Gli36ΔEGFR and U251T2 cells were authenticated via STR profiling by the University of Arizona Genetics Core via STR profiling in January 2015, and LN229 in March 2018. GBM43 were authenticated by the Cell Line Characterization Core at MD Anderson Cancer Center in February 2019. The rest cell lines were not authenticated after receipt.
Mycoplasma contamination	The cell lines were tested for mycoplasma contamination. All the cells used in this study were mycoplasma negative.
Commonly misidentified lines (See ICLAC register)	No commonly misidentified cell lines were used.

Animals and other organisms

Policy information about studies involving animals; ARRIVE guidelines recommended for reporting animal research

Laboratory animals	Six- to eight-week-old female athymic nude mice and C57BL/6 mice were purchased from Jackson Laboratories (Bar Harbor, Maine) and housed in City of Hope Animal Facility with light cycle: A 12-light/12-dark cycle and temperatures of 65-75°F (~18-23°C) with 40-60% humidity.
Wild animals	No wild animals were used in this study.
Field-collected samples	No field-collected samples were used.
Ethics oversight	Experiments and handling of mice were conducted under federal, state and local guidelines and with an approval from the City of Hope Animal Care and Use Committee.

Note that full information on the approval of the study protocol must also be provided in the manuscript.

Human research participants

Policy information about studies involving human research participants

Population characteristics	Peripheral blood cones collected from healthy donors were randomly picked. No specific age, gender or other information were considered.
Recruitment	Healthy donors donated the blood in Michael Amini Transfusion Medicine Center of City of Hope National Medical Center,
Ethics oversight	Peripheral blood cones were collected from healthy donors after written informed consent under a protocol approved by the City of Hope Institutional Review Board.

Note that full information on the approval of the study protocol must also be provided in the manuscript.

Flow Cytometry

Plots

Confirm that:

☒ The axis labels state the marker and fluorochrome used (e.g. CD4-FITC).

☒ The axis scales are clearly visible. Include numbers along axes only for bottom left plot of group (a 'group' is an analysis of identical markers).

☒ All plots are contour plots with outliers or pseudocolor plots.

☒ A numerical value for number of cells or percentage (with statistics) is provided.

Methodology

Sample preparation	For in vitro CD47 binding and blocking assays, U251T2 cells pre-blocked with 2% BSA were incubated with 0, 5, 10, 25, 50,100, 250, 500, 1000, 2500, 5000 and 10000 ng/ml purified αCD47-G1 or αCD47-G4 antibodies for 30 min. Then the cells were washed twice and stained with APC-conjugated anti-human Fc (Jackson ImmunoResearch, 209-605-098) for 20 min. After that the cells were washed twice and stained with BV786-conjunaged anti-CD47 antibody (clone, B6H12, BD, 563758) for 20 min. Median of mean fluorescence intensity (MFI) of APC and BV786 was used to determine CD47 binding and blocking capacity of αCD47-G1 and αCD47-G4.<br>For the phagocytosis assay of mouse BMDM, GBM43 and BT422 cells stained with CFSE (Thermo fisher, C34554) were used as target cells. BMDM and target cells were cocultured at a ratio of 1:2 for 2 hours in the presence of vehicle control, αCD47-G1 or αCD47-G4 at the dose of 5 μg/ml, in a humidified, 5% CO2 incubator at 37 °C in ultra-low-attachment 96-well Ubottom plates (Corning) in serum-free 1640 (Life Technologies). Then the cells were harvested by centrifuging at 400 g for 5 min at 4 °C and stained with anti-mouse CD11b (BD, 552850) to identify macrophages. For blocking Fc receptors, BMDM were pre-incubated with 10 μg/ml isotype human IgG1 (Biolegend, 403505) for 30 min. All flow cytometry data werecollected using an Fortessa X20 flow cytometer (BD Biosciences). Phagocytosis was measured as the number of CD11b +CFSE+macrophages, quantified as a percentage of the total CD11b+ macrophages.<br>For the phagocytosis assay of human primary macrophage, GBM43 cells stained with CFSE (Thermo fisher, C34554) were used as target cells. human macrophages and target cells were cocultured at a ratio of 1:2 for 4 hours in the presence of vehicle control, αCD47-G1 or αCD47-G4 at the dose of 5 μg/ml, in a humidified, 5% CO2 incubator at 37 °C in ultra-lowattachment

96-well U-bottom plates (Corning) in serum-free 1640 (Life Technologies). Then the cells were harvested by centrifuging at 400 g for 5 min at 4 °C and stained with anti-human CD11b (BD, 552850) to identify macrophages. Phagocytosis was measured as the number of CD11b+CFSE+ macrophages, quantified as a percentage of the total CD11b+ macrophages.
For flow cytometric assessment of murine immune cells, mice were euthanized at indicated time points. Mononuclear immune cells in the brain were isolated with Percoll gradient centrifugation for flow cytometric analysis.

Instrument	Fortessa X20 flow cytometer (BD Biosciences) was used to collect flow cytometric data.
Software	BD FACSDiva and FlowJo 7.6 & 10.0 were used to collect and analyze the flow cytometric data, respectively.
Cell population abundance	The purity of sorted cells was over 99%.
Gating strategy	To determine CD47 expression in GBM cells, live cells were further gated on FSC-A/SSC-A, FSC-H/FSC-W, and then SSC-H/SSCW events to check surface expression levels of CD47.<br>For CD47 binding and blocking assays, live cells were further gated on FSC-A/SSC-A, FSC-H/FSC-W, and then SSC-H/SSC-W events to check surface expression levels of CD47 and human Fc.<br>For the phagocytosis assay of macropahes, live cells were further gated on FSC-A/SSC-A, FSC-H/FSC-W, and then SSC-H/SSCW events. Macrophages were defined with CD11b(+). Phagocytosis was measured as the number of CD11b(+)CFSE(+) macrophages.<br>To determine CD69 expression in primary human NK cells, live cells were further gated on FSC-A/SSC-A, FSC-H/FSC-W, and then SSC-H/SSC-W events. NK cells were defined as CD56(+) events to check surface expression levels of CD69.<br>For flow cytometric assays on murine immune cells, live cells were gated on FSC-A/SSC-A, FSC-H/FSC-W, SSC-H/SSC-W, and then SSC-H/DAPI. NK cells were defined as NKp46(+)CD3(–) ; Macrophages were defined as CD45(high)CD11b(+)F4/80(+) ; T cells were defined as NKp46(–)CD3(+).

☒ Tick this box to confirm that a figure exemplifying the gating strategy is provided in the Supplementary Information.
